# Transcriptional signatures of the BCL2 family for individualized acute myeloid leukaemia treatment

**DOI:** 10.1186/s13073-022-01115-w

**Published:** 2022-09-28

**Authors:** Chansub Lee, Sungyoung Lee, Eunchae Park, Junshik Hong, Dong-Yeop Shin, Ja Min Byun, Hongseok Yun, Youngil Koh, Sung-Soo Yoon

**Affiliations:** 1 Cancer Research Institute, Seoul National University College of Medicine, Seoul, Republic of Korea; 2 Center for Medical Innovation, Seoul National University Hospital, Seoul, Republic of Korea; 3 Department of Genomic Medicine, Seoul National University Hospital, Seoul, Republic of Korea; 4 Center for Precision Medicine, Seoul National University Hospital, Seoul, Republic of Korea; 5 Division of Hematology and Medical Oncology, Department of Internal Medicine, Seoul National University Hospital, Seoul, Republic of Korea

**Keywords:** Acute myeloid leukaemia, B-cell lymphoma-2 family, Transcriptional signatures

## Abstract

**Background:**

Although anti-apoptotic proteins of the B-cell lymphoma-2 (BCL2) family have been utilized as therapeutic targets in acute myeloid leukaemia (AML), their complicated regulatory networks make individualized therapy difficult. This study aimed to discover the transcriptional signatures of BCL2 family genes that reflect regulatory dynamics, which can guide individualized therapeutic strategies.

**Methods:**

From three AML RNA-seq cohorts (BeatAML, LeuceGene, and TCGA; *n* = 451, 437, and 179, respectively), we constructed the BCL2 family signatures (BFSigs) by applying an innovative gene-set selection method reflecting biological knowledge followed by non-negative matrix factorization (NMF). To demonstrate the significance of the BFSigs, we conducted modelling to predict response to BCL2 family inhibitors, clustering, and functional enrichment analysis. Cross-platform validity of BFSigs was also confirmed using NanoString technology in a separate cohort of 47 patients.

**Results:**

We established BFSigs labeled as the BCL2, MCL1/BCL2, and BFL1/MCL1 signatures that identify key anti-apoptotic proteins. Unsupervised clustering based on BFSig information consistently classified AML patients into three robust subtypes across different AML cohorts, implying the existence of biological entities revealed by the BFSig approach. Interestingly, each subtype has distinct enrichment patterns of major cancer pathways, including MAPK and mTORC1, which propose subtype-specific combination treatment with apoptosis modulating drugs. The BFSig-based classifier also predicted response to venetoclax with remarkable performance (area under the ROC curve, AUROC = 0.874), which was well-validated in an independent cohort (AUROC = 0.950). Lastly, we successfully confirmed the validity of BFSigs using NanoString technology.

**Conclusions:**

This study proposes BFSigs as a biomarker for the effective selection of apoptosis targeting treatments and cancer pathways to co-target in AML.

**Supplementary Information:**

The online version contains supplementary material available at 10.1186/s13073-022-01115-w.

## Background

Acute myeloid leukaemia (AML) is an aggressive hematologic cancer of myeloid cell lineage characterized by the interruption of myeloid precursor cells’ differentiation and clonal proliferation, resulting in the accumulation of leukemic cells [1]. Despite improvements in our knowledge to help understand the biology of AML and identify potential therapeutic targets for AML [2], the overall prognosis for AML remains poor [1, 3].

The evasion of apoptosis is a hallmark of cancer [4], and the B-cell lymphoma-2 (BCL2) protein family plays a critical role in regulating intrinsic apoptosis. Depending on their functions, they are divided into pro- and anti-apoptotic proteins [5]. Expansion of abnormal cells often occurs due to the imbalance of these anti- and pro-apoptotic BCL2 family proteins and depends on specific anti-apoptotic proteins [6]. Therefore, targeting the cancer apoptosis pathway to manipulate these proteins has been rigorously explored. Among these, a combination of the BCL2-selective inhibitor, venetoclax, with conventional chemotherapeutic agents is a representative success in AML treatment [7, 8]. Despite the success of venetoclax in AML, both inherent and acquired resistance are frequently observed. One of the representative resistance mechanisms to venetoclax is attributable to MCL1, an alternative anti-apoptotic protein. Accordingly, several MCL1-selective inhibitors are under clinical investigation in AML. Likewise, BFL1, another alternative anti-apoptotic protein, has also been highlighted as a potential biomarker of venetoclax resistance in AML [9, 10]. Altogether, these emphasize the importance of identifying individual dependency on anti-apoptotic proteins for personalized medicine in AML.

Due to the distinct dual roles of proteins in intrinsic apoptosis, knowledge on their protein-protein interaction (PPI) has been accumulated from a biological perspective. For example, pro-apoptotic proteins activate mitochondrial outer membrane permeabilization, whereas anti-apoptotic proteins counteract them and inhibit apoptosis [5, 7]. PPI networks of intrinsic apoptosis have been well studied [11] and specific interactions between pro- and anti-apoptotic proteins have been discovered [7]. More importantly, well-known cancer pathways, such as TP53, NF-κB, and TGF-β pathways, are closely linked to the BCL2 family [12–20], which implies that these pathways could be potential targets for combination treatment with apoptosis modulating agents. Given the BCL2 family PPIs and their association with cancer pathways, we speculated that the transcriptional signatures reflecting the complicated regulatory networks of the BCL2 family exist and may guide individualized treatment strategies targeting the BCL2 family.

Non-negative matrix factorization (NMF) is an unsupervised approach used to extract biologically hidden meaningful signatures in the gene expression matrix [21–23]. In brief, NMF factorizes expression matrix A (*g* × *n*) into two non-negative matrices, W and H by the rank *k*, where *g* and *n* denote the number of genes and the number of samples, respectively. In this study, the *k* vectors of the W matrix summarize the gene-wise patterns, while those of the H matrix summarize the sample-wise patterns.

In high dimensional transcriptome data, data-driven feature selection can cause many false-positive genes and overlook meaningful genes due to the low signal-to-noise ratio [24, 25]. Moreover, many noise genes can arise from the various intrinsic or extrinsic factors that are dataset-specific [26–28]. To discover transcriptional signatures guiding precision treatment in AML, considering these challenges, we devised a novel approach based on establishing an innovative gene selection method, followed by the application of NMF. We hypothesized that application of NMF to identify “BCL2 family” signatures could be successful only after optimally selecting a set of genes representing the complex regulatory network of the BCL2 family. In brief, genes were selected based on well-established domain knowledge to overcome the low signal-to-noise ratio [24], followed by dataset-specific gene optimization for noise reduction using a novel approach developed by us. The optimized genes were finally processed into BCL2 family signatures (BFSig) using NMF.

Here, we present how BFSigs can efficiently classify AML into three subtypes, provide subtype-specific treatment guidance, including drug combination, and predict response to BCL2 family inhibitors. We also present the validity of BFSigs using NanoString nCounter panel in AML samples from Seoul National University Hospital (SNUH).

## Methods

### Transcriptome datasets and drug response information

We utilized multiple transcriptome datasets as follows: four patient-based RNA-seq datasets of AML [BeatAML (*n* = 451) [29], LeuceGene (*n* = 437) [9], TCGA-LAML (*n* = 179), Tavor (*n* = 43) [30, 31]] and other hematologic malignancies [TCGA-DLBC (diffuse large B-cell lymphoma; *n* = 48) [32] and CLLE-ES (chronic lymphocytic leukaemia; *n* = 111) [33]]; a cell line-based AML RNA-seq dataset [(Cancer Cell Line Encyclopedia (CCLE; *n* = 34) [34]]; and an AML NanoString dataset [35] of 47 bone marrow samples acquired from randomly chosen patients (23 of male and 24 of female from 22 to 84 of age) with AML diagnosed in Seoul National University Hospital (SNUH) during the period between April 2016 and September 2019, whose RNA was available for the experiment.

For BeatAML and LeuceGene datasets, we obtained partially available venetoclax responses from 186 and 23 samples in the original study. For Tavor dataset, we obtained venetoclax responses from 47 samples from 43 patients. We downloaded response to MCL1 inhibitors (AZD5991, MIM1, and UMI1-77; *n* = 11–12) of CCLE dataset samples from Genomics of Drug Sensitivity in Cancer2 (GDSC2). Acquisition of datasets and pre-processing of gene expression data is described in Additional file 1.

**Fig. 1:**
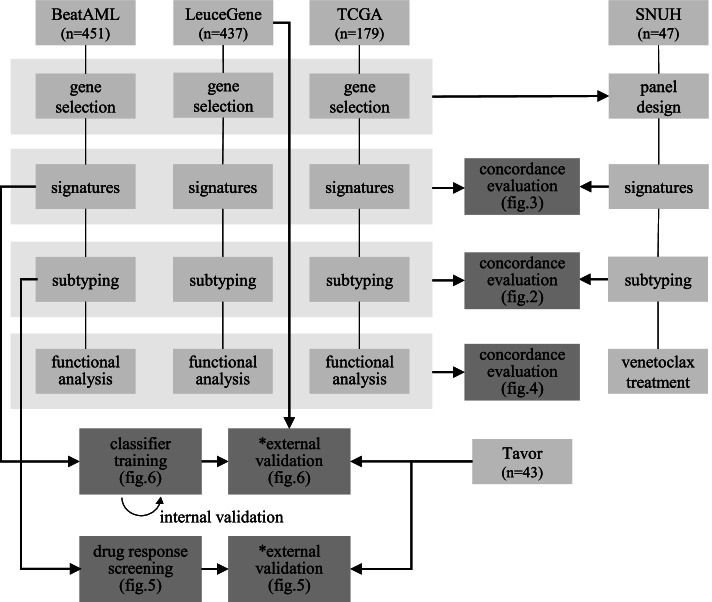
Flowchart of the study. From AML RNA-seq datasets (BeatAML, LeuceGene, and TCGA), gene optimization is independently conducted for capturing regulation factors of the BCL2 family. Afterward, the BCL2 family signatures (BFSigs) are calculated using the selected genes. Using the signatures, three novel subtypes are identified and functionally characterized. A classifier for predicting venetoclax response is developed and validated. Additionally, drug response analysis reveals the signature-based subtype-specific drug sensitivity. Finally, the validity of our selected genes is confirmed in a custom NanoString panel. *For external validation of BeatAML results, the BFSigs were re-extracted after batch effect correction

Figure 1 describes which analysis was performed with which dataset. BeatAML, LeuceGene, and TCGA-LAML datasets were used for the BFSigs discovery, subtyping, and functional analysis, and these results were compared with those of other hematologic malignancy datasets (TCGA-DLBC and CLLE-ES). Using BeatAML, LeuceGene, and Tavor datasets whose subsets have drug response information, we searched BFSigs-associated drugs and developed a venetoclax response classifier. Additionally, we analysed predicting response to MCL1 inhibitor using GDSC2-availble CCLE samples. NanoString dataset was used for cross-platform validation for the BFSgis, and a subset of 7 samples was subjected to a venetoclax assay.

### Selection of genes that reflect the regulation network of the BCL2 family

In this study, we propose novel transcriptional signatures reflecting the regulation network of the BCL2 family. The BFSigs were calculated in two steps: (1) gene collection and (2) NMF-based gene optimization (Additional file 1). In brief, for the gene selection step, we identified genes closely related to the BCL2 family from a curated gene-set database [36–62] (Additional file 1). In each AML dataset, subsequently, a backward selection was conducted to remove high-noise genes with a postulation that they may not be helpful in the imputation of the profiles of the BCL2 family genes (BCL2, MCL1, BFL1, BCLXL, and BCLW) [63, 64] (Additional file 2: Fig. S1). In this respect, we sought to minimize the imputation error by reconstructing their profiles using NMF with datasets simulated with missing BCL2 family gene expression (*NA* value in R).

### Calculation of the transcriptional signature of BCL2 family

Optimized genes that minimized the imputation error were finally processed into BFSigs using NMF. The calculation was performed using the NNLM (ver. 0.4.3) R package with the loss function as mean Kullback-Leibler divergence [65]. For optimal rank selection in NMF, we used a cophenetic correlation coefficient which reflects the stability of sample clustering for multiple ranks, using the NMF (v0.21.0) R package [66]. Moreover, a detailed consensus matrix was obtained from the connectivity matrices of the repeatedly calculated H matrix using the cophenetic correlation coefficient. Finally, we selected the rank in which the cophenetic correlation coefficient begins to fall.

To confirm the validity of BFSigs, we compared them between venetoclax response groups in addition to the original expression of the BCL2 family using Welch’s *t*-test. To avoid bias from multiple samples from the same patient, only those samples with the latest time point were chosen when the samples were assigned to the same response group (Additional file 3: Table S1).

### Identification of AML subtypes based on the signatures

To identify the subtype defined by BFSig, we conducted hierarchical clustering with average linkage and Pearson correlation distance. Visualization of clustering and profiling signatures was conducted using the pheatmap (v1.0.12) R package.

We compared the number of samples included in the subtypes using the chi-square test. For identifying subtype-wise pathway enrichments, we performed gene set enrichment analysis (GSEA) using fgsea (v1.10.1) [67]. In this study, the following pathways of MsigDB (v7.0) were considered: KEGG, GO, hallmark, BioCarta, Reactome, and PID [68]. The result of GSEA was derived as the normalized enrichment score (NES) and its *p*-value. In this step, we applied two types of NES calculation (one vs. others, one vs. one).

### Drug response screening across BCL2 family signature subtypes

To explore the drugs that act differently across the identified BFSig-based subtypes, we analysed the responses of 122 drugs available from the BeatAML dataset. We analysed associations between BFSigs subtypes and the area under the drug response curve (AUC) using Kruskal-Wallis test and Wilcoxon rank-sum test. Validation was performed using Tavor dataset in which the area above the drug response curve (ACC) was available.

### Classifier for predicting venetoclax response

We developed a binary classifier using BeatAML, which predicts the response to venetoclax using BFSig. Performance evaluation was repeated 10 times to avoid overfitting. For each run, 70 and 30% of the samples were randomly assigned to train and test sets, respectively. We developed a logistic regression model with the train set and evaluated this model with the test set. For each sample, a probability of sensitivity was calculated by averaging the predicted probability from the 10 times repeated training-testing scheme. Additionally, we computed the area under the ROC curve (AUROC) by combining the predicted probabilities of all repeats, and its significance was tested with two-sided DeLong’s test using the pROC R package (v 1.16.2). Response groups were separated by binarizing them as sensitive if IC_50_ ≤ 1 μM and resistant if IC_50_ ≥ 10 μM (Additional file 2: Fig S2A). The classifier was compared with other variables: original expression of (1) three anti-apoptotic proteins BCL2, MCL1, and BFL1, (2) former genes plus BCLXL, and BCLW, and (3) top 5, 10, 30, and 50 differential expression genes (DEGs), which were estimated based on the fold change between the sensitive and resistance groups. Additionally, other machine learning methods were compared: (1) Lasso logistic regression, (2) random forest, and (3) support vector machine using the sklearn (v0.20.4) package in python 3.4.9.

We performed an external validation using LeuceGene and Tavor datasets. For Tavor dataset, the response groups were separated as in BeatAML (Additional file 2: Fig S2B). For LeuceGene dataset, we used the binary response group information provided in the original study [9]. After batch effect correction and gene optimization, the BFSigs were re-extracted from the merged data (Additional file 1). The sensitivity probabilities of samples in LeuceGene and Tavor datasets were calculated using a classifier re-trained using whole samples of BeatAML dataset.

### Classifier for predicting MCL1-selective inhibitor response

We used 34 AML cell line RNA-seq data from CCLE and matched drug response (AUC; area under the dose-response curve) of BCL2 family inhibitors from GDSC2. Due to an insufficient number of samples, we extracted the BFSigs for cell lines from the merged data of BeatAML and CCLE. After correcting the batch effect and optimizing genes, we extracted the BFSigs (Additional file 1). Afterward, we measured prediction performance for the inhibitory response. Due to a small number of samples (*n* = 11 or 12), we did not separate test sets and instead used LOOCV (Leave-One-Out Cross-Validation). Because the sample sizes were too small to apply the sensitive and resistant group thresholds for binary classification, we used a linear regression model for predicting drug’s AUC. NRMSE (Normalized Root Mean Square Error) was calculated to measure the performance.

## Results

### Outline of study

The overall workflow of the study is described in Fig. 1. First, from public RNA-seq datasets [BeatAML (*n* = 451), LeuceGene (*n* = 437), and TCGA (*n* = 179)], gene selection was conducted for capturing regulation factors of the BCL2 family. We first collected genes related to BCL2 family regulation from an extensive collection of domain knowledge. Subsequently, we optimized the above genes to filter out noise independently for each dataset using our novel NMF-based approach. Second, the optimized genes were processed into dataset-specific BFSigs using NMF. Using the BFSigs, we identified three AML subtypes and conducted functional enrichment analysis for each subtype. These analyses showed remarkable concordance results. In the subtypes, we discovered subtype-specific drug sensitivity. Moreover, we trained a simple classifier to predict venetoclax response, then evaluated its performance via internal and external validation. We designed an expression panel to validate our BFSigs in extracting signatures and subtyping an independent SNUH AML cohort with the selected genes.

### Construction of highly concordant BFSigs

First, we collected 236 genes related to BCL2 family regulation from multiple curated gene-set databases and literature [36–62] (Additional file 1). Next, utilizing NMF-based imputation, we optimized the gene collection to filter out genes that do not contribute to the imputation of the BCL2 family genes that are set to the missing values in the simulated datasets (Additional file 2: Fig S1A; details in “Methods”). As a result, 97, 107, and 127 genes were chosen for BeatAML, LeuceGene, and TCGA, respectively (Additional file 2: Fig S1B and Additional file 3: Table S2). Interestingly, up to 84.5% of genes were commonly identified in at least two datasets (84.5, 83.2, and 63.0% for BeatAML, LeuceGene, and TCGA, respectively), which reflects high concordance of the proposed approach.

After gene selection, to extract BFSigs, we decomposed the expression matrix of the optimized genes from each dataset into two matrices that represent summarized patterns of genes (W) and samples (H), respectively (Additional file 4: Table S3). We determined the optimal rank of these matrices, and therefore the number of signatures, as three, and confirmed that the optimal rank is identical across all the datasets (Additional file 2: Fig S3). With the putative venetoclax- resistant factors (BCL2, MCL1, and BFL1; identified in Additional file 2: Fig S4), we classified each BFSig as BCL2, MCL1/BCL2, or BFL1/MCL1 signatures, then performed interpretation.

**Fig. 2:**
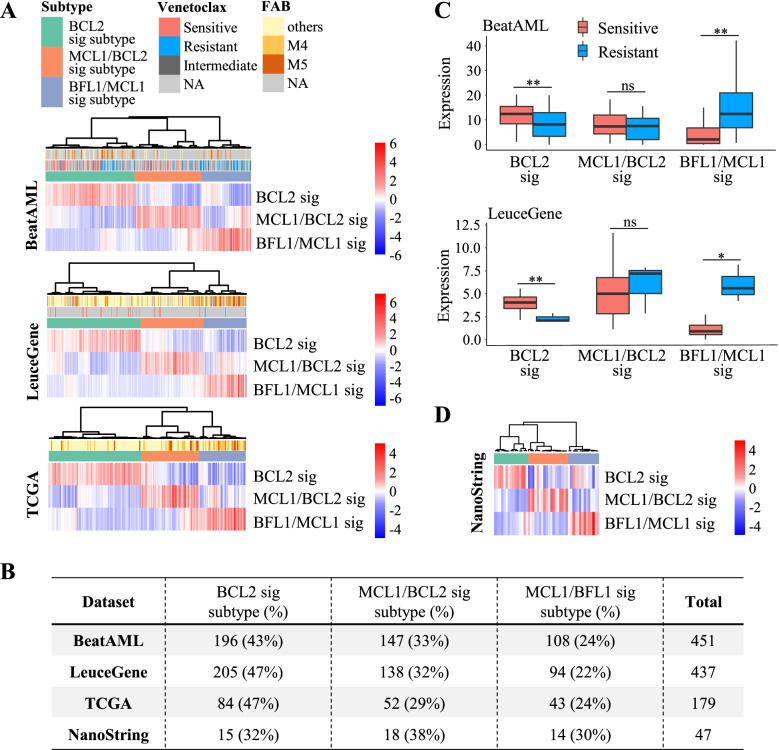
Identification of BCL2 family-based acute myeloid leukaemia (AML) subtypes. **A** Profiles of BCL2 family signatures calculated using optimized genes in each RNA-seq dataset (BeatAML, LeuceGene, and TCGA). These datasets show three distinct clusters annotated as BCL2, MCL1/BCL2, and BFL1/MCL1 signature subtypes. Columns are clustered using hierarchical clustering with average distance. **B** Sample proportion of these subtypes. **C** Comparison of BCL2 family signatures between venetoclax response groups in BeatAML (81 sensitive and 72 resistant) and LeuceGene (20 sensitive and 3 resistant). *P*-values are calculated by Welch’s *t*-test. * < 0.05, ** < 0.01, ns > 0.10. **D** A profile of BCL2 family signatures in the NanoString dataset. The samples are also divided into three clusters resulting from RNA-seq datasets

**Fig. 3:**
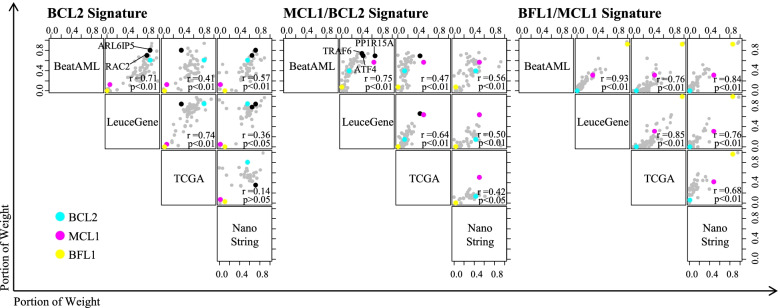
Concordance of BCL2 family signatures between acute myeloid leukaemia (AML) datasets. Weight of optimized genes in the definition of the BCL2 family signatures (BCL2, MCL1/BCL2, and BFL1/MCL1 signature). BCL2, MCL1, and BFL1 are marked in cyan, magenta, and yellow, respectively. Some determinant components of the signatures are marked in black. Four AML datasets show the consistent weight of optimized genes. Each weight of genes is normalized to sum 1. Correlation coefficients are calculated using Spearman’s rho

Interestingly, all of the AML datasets showed consistent patterns of the BFSigs (H matrix), as shown in Fig. 2A. Moreover, our identified BFSigs showed that the optimal genes consistently contributed to each signature across three datasets (Fig. 3; Spearman’s rho = 0.41–0.93 between BeatAML, LeuceGene, and TCGA). Especially, the putative venetoclax-resistant factors (BCL2, MCL1, and BFL1) consistently dominated the BFSigs (Additional file 2: Fig S5A; Spearman’s rho = 1). In the other hematologic malignancies, on the other hand, these genes showed inconsistent contribution to the signatures, which results in different signatures from those of AML (Spearman’s rho −0.31–0.29, *p* > 0.05), except the BCL2 signature of chronic lymphocytic leukaemia (Spearman’s rho 0.59, *p* < 0.01) (Additional file 2: Fig S5 and S6A). These results suggest that the BFSigs are heterogeneous across hematologic malignancies.

In addition, we identified not only BCL2 family genes but also other optimized genes with comparable roles in closely regulating BCL2 family genes, such as ARL6IP5 and RAC2 in the BCL2 signature and TRAF6, ATF4, and PP1R15A in the MCL1/BCL2 signature (Fig. 3). These findings implied that the BFSigs reflect the regulation link between BCL2 family genes and their specific co-contributing genes (explained in the “Discussion” section).

### Signature-based AML subtyping shows distinct pathobiology among AML

Our signature analysis of the three AML datasets revealed that the AML samples could be classified into three molecular subtypes (Fig. 2A). We annotated these subtypes as BCL2 signature, MCL1/BCL2 signature, and BFL1/MCL1 signature subtypes based on the dominantly expressed signature in each subtype. The sample proportion of subtypes was not different across datasets (Fig. 2B, chi-square *p* = 0.51), meaning that the BFSigs-based AML subtypes are consistently distributed regardless of the datasets. In both BeatAML and LeuceGene datasets, the venetoclax-resistant group showed significantly lower BCL2 signature and higher BFL1/MCL1 signature than the venetoclax-sensitive group (Welch’s *t*-test *p* < 0.01 and *p* < 0.05, respectively; Fig. 2C).

**Fig. 4:**
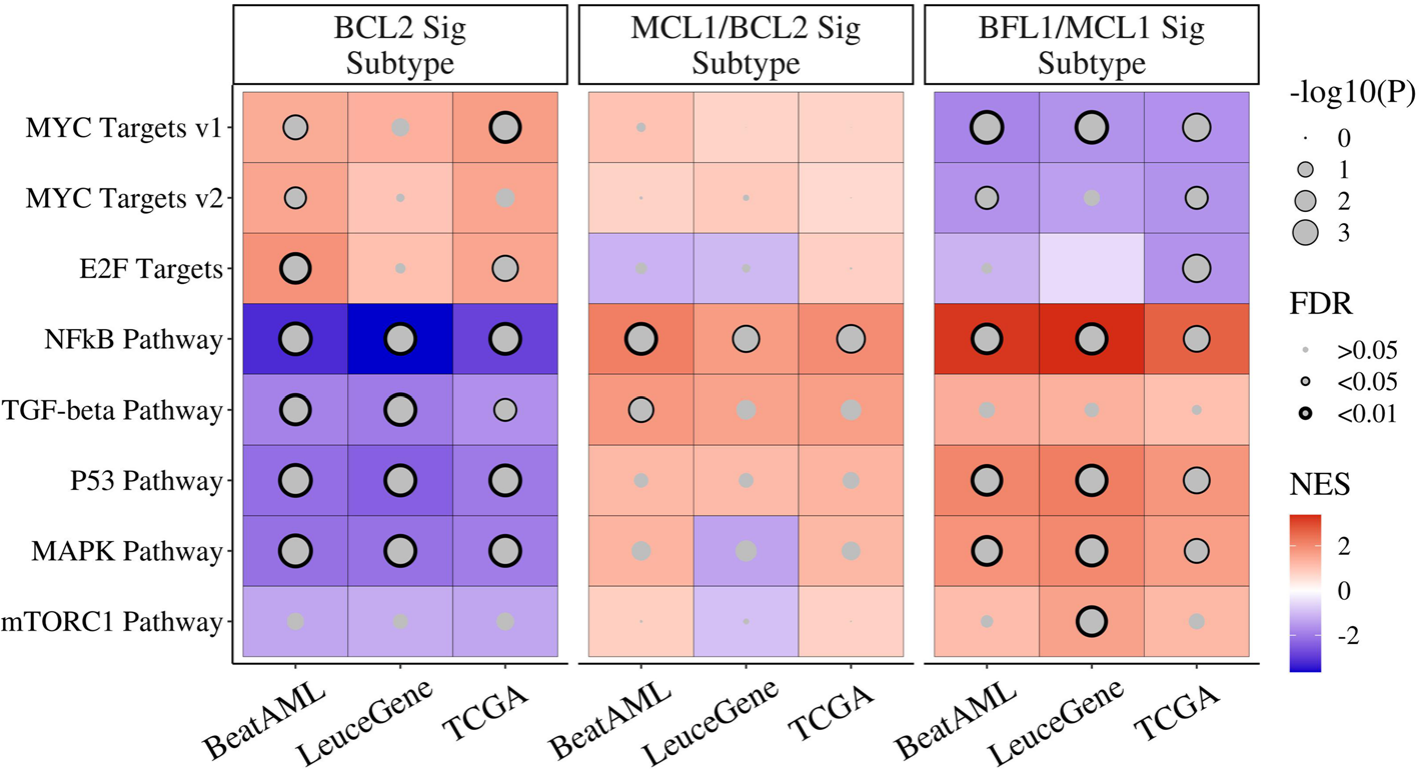
Functional analysis of BCL2 family-based acute myeloid leukaemia (AML) subtypes. Gene set enrichment analysis (GSEA) from the comparison between one subtype and the others identifies enriched gene sets in each subtype. Enrichment patterns are consistent across three AML datasets. NES indicates a normalized enrichment score. The gene set of the MAPK pathway is from the GO database. The others are from the hallmark database

From the GSEA results, we discovered that the signature-based subtypes manifest distinct enrichment patterns (Fig. 4 and Additional file 5: Table S4), which explain their biological characteristics. BFL1/MCL1 signature subtype showed significant enrichment of NF-κB pathway, p53, mitogen-activated protein kinase (MAPK), and mammalian target of rapamycin complex 1 (mTORC1) pathways (FDR < 0.05); MCL1/BCL2 signature subtype presented significant enrichment of the NF-κB and TGF-β pathways (FDR < 0.05); the BCL2 signature subtype was linked to upregulation of Myc and E2F target genes (FDR < 0.05). Of course, other hematologic malignancies showed different enrichment results in these pathways (Additional file 2: Fig S6B). Additional mutation analysis showed no significant difference among subtypes in AML (Additional file 2: Fig S7).

### Relationship between BFSigs and sensitivity to mTORC1 and MAPK pathway inhibitor

**Fig. 5:**
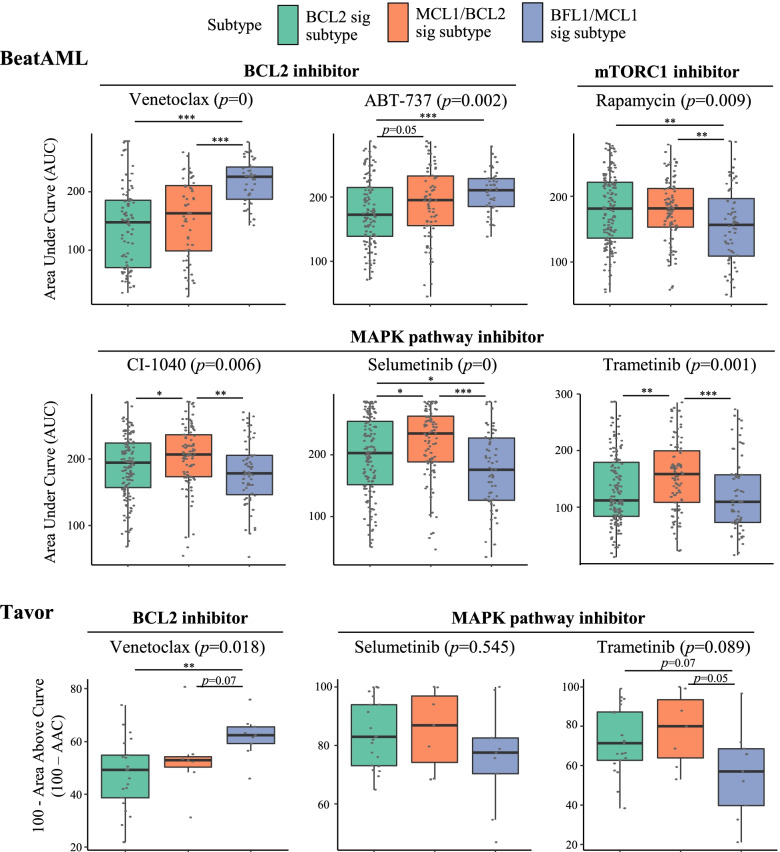
Association between BFSigs and drug responses. Comparison of drug responses between the subtypes in BeatAML and Tavor datasets. The lower the *y*-axis value, the more sensitive to the drugs. *P*-values above the panel and between the box plots are calculated by Kruskal-Wallis test and Wilcoxon rank-sum test, respectively. * < 0.05, ** < 0.01, *** < 0.001, ns > 0.05

The drug screening analysis revealed that not only venetoclax but also other drugs showed subtype-specific sensitivity. In BeatAML dataset, BFL1/MCL1 subtype was more sensitive to rapamycin, an inhibitor of mTORC1, than other subtypes (*p* < 0.01) (Fig. 5 and Additional file 2: Fig S8). Blocking MAPK pathway was also more effective in BFL1/MCL1 subtype (*p* < 0.05). Especially, the result of trametinib, an inhibitor of MEK, was validated in Tavor dataset (*p* < 0.07) (Fig. 5 and Additional file 2: Fig S9). These results were in line with the GSEA results in which mTORC1 and MAPK pathways were enriched in BFL1/MCL1 subtype samples.

### BFSigs and prediction of response to apoptosis modulating anticancer drugs

**Fig. 6:**
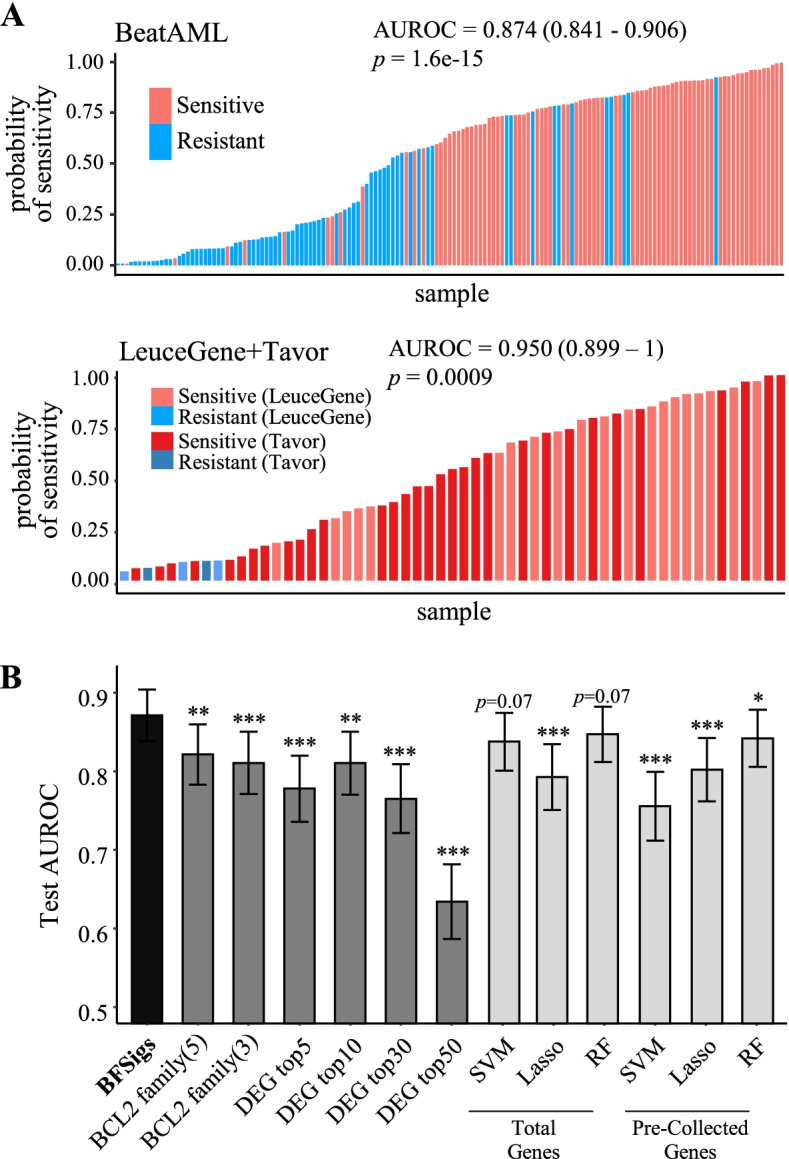
Prediction response to venetoclax. **A** Probability of sensitivity to venetoclax calculated from BCL2 family signature-based logistic regression model in training set (BeatAML; 81 sensitive and 72 resistant) and external validation set (LeuceGene and Tavor; 20+32 sensitive and 3+2 resistant). The sensitivity probabilities of BeatAML represent the average probability from the 10-times repeated training-testing scheme. Those of LeuceGene and Tavor are calculated from the whole BeatAML-based classifier. *P*-values are calculated using Wilcoxon rank-sum test by comparing the probability rank between the response groups. **B** Comparison of prediction performance between venetoclax response classifiers. The black bar indicates the BCL2 family signature-based logistic regression model. The dark grey bars indicate logistic regression models using the original expression of five BCL family genes (BCL2+MCL1+BFL1+BCLXL+BCLW), three BCL2 family genes (BCL2+MCL1+BFL1), and top differentially expressed genes (DEGs), respectively. The light grey bars indicate machine learning-based models using total genes or pre-collected genes related to BCL2 family regulation. The used machine learning methods are support vector machine, Lasso, and random forest (RF). Error bar indicates 95% confidence interval (CI). *P*-values are calculated compared with the signature model using DeLong’s test. * < 0.05, ** < 0.01, *** < 0.001

After excluding duplicated samples from same patient in the same response group, 153 samples (81 sensitive and 72 resistant) and 34 samples (32 sensitive and 2 resistant) were used in the BeatAML and Tavor analysis, respectively (Additional file 3: Table S1). Using the BFSigs, we built a classifier for predicting venetoclax response using BeatAML (*n* = 153). From internal validation using our 10-times repeated training-testing scheme, our signature-based classifier outperformed other classifiers using original expression- or machine learning-based approaches (Fig. 6A, B). First, our signature-based classifier using logistic regression achieved an AUROC of 0.874 in the testing dataset (95% CI 0.841–0.906), which was substantially superior to the classifiers using the original expression of the anti-apoptotic BCL2 family or DEGs (AUROC 0.634–0.822, *p* < 0.002). Furthermore, our signature-based classifier showed better performance (*p* < 0.05 except for two models with *p* < 0.07) in comparison to three machine learning methods (support vector machine, lasso, and random forest; AUROC 0.756–0.847). In the external validation using the batch-corrected LeuceGene and Tavor datasets (*n* = 23 + 34) (Additional file 2: Fig S10A and B), the signature-based classifier trained with whole BeatAML also showed improved performance (AUROC = 0.950, 95% CI 0.899–1) relative to all other approaches (AUROC 0.562–0.912), with the exception of five anti-apoptotic BCL family gene-based approach (AUROC = 0.943, 95% CI 0.887–1) (Fig. 6A and Additional file 2: Fig S11). DeLong’s test was not conducted because of the small number of resistant samples (*n* = 5) in the external validation set.

Here, we confirmed the possibility that BFSig can be applied in predicting drug response to not only BCL2 inhibitors but also MCL1 inhibitors (UMI-77, MIM1, and AZD5991). To extend the proposed approach to MCL1 inhibitors, we further extracted the signatures from the batch-corrected CCLE dataset (Additional file 2: Fig S10C and S12; Additional file 4: Table S3). From the linear regression model for predicting AUC (area under the dose-response curve) (Additional file 6: Table S5), UMI-77 was more predictable (LOOCV NRMSE = 0.36) when using the BFSigs rather than using an original expression. In AZD5991, MIM1, and venetoclax, the BFSigs showed similar performance with three BCL2 family genes (BCL2+MCL1+BFL1) (Additional file 2: Fig S13).

In methodological view, we showed that the gene optimization and rank selection significantly enhanced prediction power in BeatAML (*p* < 0.05) (Additional file 2: Fig S14); AUROCs of the non-optimized NMF-based signature and PCA (principal component analysis)-based signature were 85.0 and 82.3, respectively. The prediction power was decreased when the signatures were calculated using optimized genes in other datasets (LeuceGene or TCGA) or intersection of the optimized genes in the three datasets (Additional file 2: Fig S1B and S15). These results emphasize the effect of dataset-specific gene optimization.

### Cross-platform validation of BFSigs

We designed a custom NanoString nCounter expression panel with our 82 optimized genes and conducted an independent study using 47 AML samples from SNUH. The 82 target genes were selected with criteria of optimal genes in BeatAML and additionally in LeuceGene or TCGA (Additional file 2: Fig S1B). Among these 82 genes, our optimization algorithm yielded 50 genes (Additional file 3: Table S2) and three BFSigs were calculated (Fig. 2D and Additional file 4: Table S3).

The additional study showed that the BFSigs can be successfully reproduced using the NanoString platform in an independent cohort despite using different sequencing technology. The optimal rank and the number of subtypes in the NanoString dataset were identical to those of RNA-seq datasets, showing the consistence of BFSigs (Additional file 2: Fig S3, Fig. 2B, D). In the BFSigs of the NanoString dataset, in addition, the weight of genes showed significantly correlated with those from the three development RNA-seq datasets (Spearman’s rho = 0.36–0.84, *p* < 0.05), especially on the anti-apoptotic proteins, except the BCL2 signature of TCGA dataset (Spearman’s rho = 0.14, *p* > 0.05) (Fig. 3). The BCL2 signature subtype showed higher sensitivity to venetoclax than other subtypes, in a subset of 7 samples from the NanoString dataset subjected to an apoptosis assay (Additional file 2: Fig S16). These results confirmed the validity of our gene signatures in extracting BFSigs and subtyping AML.

## Discussion

In this study, we discovered three BFSigs whose key proteins were BCL2, MCL1/BCL2, and BFL1/MCL1, respectively in AML. The combination of the non-biased NMF approach with a novel gene selection method based on biological knowledge gave birth to BFSigs with biological and clinical relevance. We could validate the robustness of suggested BFSigs from biological, statistical, and clinical viewpoints. First, the BFSigs successfully and consistently divided the samples from multiple and independent AML datasets into three subtypes. Second, our imputation-based noise reduction approach for resolving the heterogeneity showed its performance and suggested its broad applicability. Third, the BFSigs demonstrated both its prediction power that outperforms the conventional marker-based approaches and its robustness from a cross-platform validation study. Accordingly, BFSigs revealed the underlying biology of BCL2 family proteins and suggested clinical utility for AML patient care.

BFSigs reflect the regulation network of the BCL2 family as a result of optimal utilization of the established domain knowledge with our novel approach. Notably, we could confirm the biological relevance of our BFSigs: (1) RAC2 and ARL6IP5, which are well-known regulators of BCL2, were the main determinants of BCL2 signature [53, 69, 70]. (2) Similarly, among the MCL1/BCL2 signature’s determinants, PPP1R15A, and TRAF6 are involved in MCL1 stabilization [71] and another determinant, ATF4, regulates the transcription of NOXA, a strong selective antagonist of MCL1 [72]. (3) The BFL1/MCL1 signature was enriched in monocytic AMLs that have been reported to be resistant to BCL2 inhibition (Additional file 2: Fig S17) [73, 74]. This signature was also highly correlated with an existing monocyte signature correlated with BFL1 and MCL1 expression and venetoclax resistance (Additional file 2: Fig S18) [75].

From a clinical perspective, BFSigs classified AML into three subtypes, with the identification of key anti-apoptotic proteins. The classification was consistent in three AML cohorts with high similarity, indicating the robustness of this discovery. Therapeutically, as the BCL2 signature subtype that shows sensitivity to BCL2 inhibitors, as observed in this study, we can assume that the MCL1/BCL2 signature subtype might be sensitive to dual inhibition of MCL1 and BCL2. In fact, based on in vivo studies, several clinical trials have utilized dual inhibition of MCL1 and BCL2 in BCL2 inhibitor-resistant AML [76]. Likewise, although there are no effective methods for dual inhibition of BFL1 and MCL1 thus far, it is clear that both proteins are associated with resistance to BCL2 inhibitors. Based on recent development of a dual inhibitor targeting them, we encourage studies on the BFL1/MCL1 signature subtype [77]. Based on our results, an umbrella trial utilizing BCL2, MCL1/BCL2, and BFL1/MCL1 inhibitors based on BFSigs seems to be an ideal design to maximize the success of apoptosis inducing agents in AML [78].

It is also worth focusing on the observations that specific cancer pathways are enriched in AML subtypes based on BFSigs. The cancer pathways, including P53, NF-κB, TGF-β, MYC, and E2F, are well-known regulators of the BCL2 family, but little is known about the exact control mechanisms [12–19, 47, 79–81]. Subtype-specific enrichment of these pathways per BFSig-based subtype suggests each BCL2 family protein has specific regulation mechanisms. In addition to the further dissection of biological mechanisms, our findings have clinical value. For example, MAPK and mTORC1 pathways are enriched in the BFL1/MCL1 signature subtype (Fig. 4), and drug response screening analysis shows that blocking these pathways is effective in the BFL1/MCL1 signature subtype (Fig. 5 and Additional file 2: Fig S8). Hence, pathway-level characterization of the BFSigs in our study could be utilized to develop novel treatment strategy in AML per subtype, such as combining inhibition of the determinant proteins and the enriched pathways.

Finally, we could confirm the potential for utilization of BFSigs in AML patient care from a cross-platform validation study using the NanoString platform. As our results suggest, the clinical utility of BFSigs in several perspectives, including umbrella trials, we endeavour to investigate the possibility of clinical application of BFSigs. While it is well-known that although whole transcriptome sequencing (WTS) contains plentiful information enabling novel research, it is difficult to standardize, limiting its clinical application. Accordingly, we validated BFSigs using the NanoString platform, which has already been adopted in clinical practice.

However, there is more to be studied based on our results. Of note, it is necessary to study MCL1 or BFL1 inhibition further. We only identified MCL1 or BFL1-related signatures and discovered that some patients are enriched with these signatures. However, it is uncertain if using the signatures could ultimately be associated with the inhibitory effect of MCL1 or BFL1 as we could not analyse these due to a lack of inhibitors for BFL1. It is also a limitation that our analysis on MCL1 inhibitors was based on a small number of cell lines due to a lack of clinical data with MCL1 inhibitors. In addition, there was heterogeneity in each BFSig-based subtype (Fig. 2A), especially in BFL1/MCL1 signature subtype. It requires additional investigation to identify their role in the future.

## Conclusions

In summary, we successfully overcame data discrepancies between multiple RNA-seq datasets and between platforms by unsupervised filtering of noise genes not related to the BCL2 family. Our approach replicated the results of extraction signatures and signature-based subtyping from the RNA-seq datasets, using an independent AML cohort and technology (NanoString). Based on our study’s clear and consistent results, we suggest BFSigs as easy-to-calculate biomarkers for effective selection of strategy in manipulating the BCL2 family in individualized treatment.

## Supplementary Information

**Additional file 1: Supplementary methods.**

**Additional file 2: Fig S1.** Gene optimization. **Fig S2.** Histogram of venetoclax IC50 values from the BeatAML dataset. **Fig S3.** Optimal rank selection for NMF. **Fig S4.** Association between the BCL2 family and venetoclax response. **Fig S5.** Contribution of the BCL2 family to signatures. **Fig S6.** Identification of BCL2 family-based subtypes in other hematologic malignancies. **Fig S7.** Mutation status of BCL2 family-based subtypes. **Fig S8.** Comparison of BCL2 family-based acute myeloid leukaemia (AML) subtypes based on drug response. **Fig S9.** Assignment of BFSig subtypes in Tavor Dataset. **Fig S10.** Batch effect correction. **Fig S11.** External validation of BCL2 family signature-based classifier. **Fig S12.** Profile of BCL2 family signatures in acute myeloid leukaemia (AML) cell lines. **Fig S13.** Prediction response to BCL2 family inhibitors in cell lines. **Fig S14.** Improvement of Prediction Power via Gene Optimization and Rank Selection. **Fig S15.** Prediction Power using Optimized Genes in External Datasets. **Fig S16.** Venetoclax Response in NanoString Samples. **Fig S17.** Relationship between BFL1/MCL1 signature and FAB classification of AML. **Fig S18.** Correlation between BCL2 family signatures and monocyte signatures. **Fig S19.** Scheme of gene optimization algorithm. **Fig S20.** Performance Measurements in Imputation of BCL2 family Profiles.

**Additional file 3: Table S1.** Samples Used in Classifier. **Table S2.** Optimized Genes.

**Additional file 4: Table S3.** NMF Matrices.

**Additional file 5: Table S4.** Gene Set Enrichment Analysis.

**Additional file 6: Table S5.** Prediction Performance in Cell Line Dataset. **Table S6.** NanoString Codeset. **Table S7.** Normalized Counts of NanoString Samples. **Table S8.** Pre-Collected Genes.

## Data Availability

Availability of public RNA-seq data is described in Additional file 1. For NanoString dataset is available in Gene Expression Omnibus (GEO) with accession number GSE209822 (www.ncbi.nlm.nih.gov/geo/query/acc.cgi?acc=GSE209822) [35]. The optimization algorithm script in the study is deposited in github (https://github.com/cslee159/OptimalGeneNMF) [82].

## Abbreviations

BCL2: B-cell lymphoma-2
AML: Acute myeloid leukaemia
NMF: Non-negative matrix factorization
NES: Normalized enrichment score
DEGs: Differentially expressed genes
AUC: Area under the drug response curve
AUROC: Area under the ROC curve
