# Supplementary material for: Transcriptional signatures of the BCL2 family for individualized acute myeloid leukaemia treatment
Source: Genome Med. 2022 Sep 28;14:111. doi: 10.1186/s13073-022-01115-w (PMC9520894; doi:10.1186/s13073-022-01115-w)
Supplement: Supplementary file 1 — Additional file 1: Supplementary methods. [file 13073_2022_1115_MOESM1_ESM.docx]

**Supplementary Methods**

***Sequencing Datasets***

For the BeatAML dataset, the Reads Per Kilobase of transcript per Million mapped read (RPKM) values obtained from RNA-seq data of 451 samples and partially available venetoclax response of 186 samples were acquired from the original study ^1^. For the LeuceGene dataset, accession numbers GSE49642, GSE52656, GSE62190, GSE66917, and GSE67039 were downloaded from Gene Expression Omnibus (GEO) to obtain the RNA-seq dataset of 437 patients, in addition to partially available venetoclax response of patients (*n* = 23) from the original study ^2^. For the TCGA dataset, TCGA-LAML (n = 179) and TCGA-DLBC (n = 48), RNA-seq raw data were downloaded from GDC Legacy Archive, in addition to RNA-seq data of CLLE-ES (n = 111) from the ICGC data portal. For the Tavor dataset, we downloaded the expression matrix and the venetoclax response of 43 patients from ORCESTRA (<https://doi.org/10.5281/zenodo.4585705>) ^3^. Raw RNA-seq data of cell line from CCLE and drug’s AUC information were obtained from sequence read archive (SRA) with accession number PRJNA523380 and genomics of drug sensitivity in cancer 2 (GDSC2), respectively.

For the SNUH cohort, we sequenced using a custom-designed NanoString nCounter gene expression code set for target genes (**Additional file 6: Table S6**), including the 82 genes constructing the BFsigs (**Additional file 3: Table S2**) and 6 housekeeping genes (B2M, GAPDH, SDHA, TBP, TPT1, and UBB) identified in this study. We checked RNA integrity using Agilent 2100 BioAnalyzer (Agilent Technologies, CA, USA) with RNA integrity number being greater than six. For each gene, probes were screened for hybridization efficiency, the potential for cross-hybridization, and GC content. Finally, the best scoring probe was selected. Hybridization reactions were performed according to the manufacturer’s protocol.

***Pre-processing of Sequencing Datasets***

The BeatAML dataset is provided as RPKM values ^1^. The other RNA datasets were processed as follows. Alignments were conducted using the Subread aligner (ver. 1.5.0-p2) ^4^ on human GRCh37 build reference genome from the Genome Analysis Toolkit (GATK) resource bundle. Read counts were measured using featureCounts (ver. 1.5.0-p2) ^5^ with Ensembl release-75. All genes with mapped read counts less than 10 across the same or more than 90% of samples were removed for each dataset. In case multiple ensembl genes were matched to one gene symbol, we first adopted the ensembl genes appearing in the BeatAML matrix, otherwise, those with higher variance were adopted. The RPKM was finalized after conditional quantile normalization ^6^ that considers GC-content correction.

For the NanoString dataset, raw counts data were generated using the nCounter analyser. They were imported into the nSolver software developed by NanoString for quality control review. Normalization was conducted based on the nSolver user manual (<https://www.nanostring.com/>) using the housekeeping genes as reference (**Additional file 6: Table S7**).

***Collection of BCL2 Family-Related Genes***

We collected genes transcriptionally-, regulationally- or functionally-related with the BCL2 family from a curated gene-set database, Molecular Signatures Database (MsigDB v6.2) ^7^ and GeneGo (<https://portal.genego.com/>). BCL2 family regulation-related genes were also added to construct the initial candidate genes (denoted as pre-collected gene set in this study) through a literature curation. All sources of the pre-collected gene set are described in **Additional file 6: Table S8**.

***Optimization Genes Algorithm to Extract BCL2 family Signatures***

An overall scheme of our gene optimization is depicted in **Additional file 2: Fig S19**. In order to select a dataset-specific optimal gene set that represents transcriptional signatures of the BCL2 gene family, a backward selection was performed with the pre-collected gene set in each dataset. As a result, a subset of "pre-collected" genes was selected so as to optimally impute the expression profiles of BCL2 family genes (BCL2, MCL1, BFL1, BCLXL, and BCLW).

In this study, to evaluate imputation power, we constructed 10 sets of simulated missing datasets in which five BCL2 family genes of each fold in 10-fold split samples were set to missing value (set to *NA* in R) (**Additional file 2: Fig S20**). After applying NMF to the each simulated missing dataset, their results were multiplied again to produce a “imputed matrix” whose missing values were imputed by NMF. In each imputed matrix, mean absolute percentage error (MAPE) of BCL2 family genes was calculated by comparing it with those in the original matrix. We used an average of mean absolute percentage errors (aMAPE) from 10 imputed matrices to measure imputation accuracy.

Using aMAPE as a selection criterion, we performed a backward selection with the pre-collected genes. For each round, genes except for the BCL2 family genes were individually eliminated to calculate aMAPE after removal of each gene, and the genes with minimal aMAPE were removed. This process was repeated until the number of genes reached 20, and we called the genes with minimal aMAPE “optimal genes”. Subsequently, we extracted the BFSigs using NMF from the expression matrix consisting of optimized genes. In outcomes of NMF, the W matrix represents the coefficient of the optimized genes in BFSigs and the H matrix represents the BFSigs profile of the samples. The optimization algorithm script is available in <https://github.com/cslee159/OptimalGeneNMF> ^8^. NMF calculation was performed using the NNLM (ver. 0.4.3) R package with the loss function as mean Kullback-Leibler divergence ^9^.

***Combining Multiple Datasets***

Before analysing multiple datasets together, we corrected the batch effect using the removeBatchEffect function of the limma R package ^10^. To visualize the existence of batch effect between datasets, we performed Principal Component Analysis (PCA). Tavor and CCLE datasets were combined with BeatAML to overcome an insufficient number of samples to extract BFSigs. When to validate the classifier trained with BeatAML, BFSigs were re-extracted from the merged data of BeatAML+LeuceGene and BeatAML+Tavor, respectively.

***Drug Response Screening across BCL2 Family Signature Subtypes***

For 122 drugs available from the BeatAML dataset, IC50 values were binarized to sensitive and resistance groups as in the venetoclax groups. We compared the number of samples included in the three subtypes using the chi-square test between the groups.

***Monocyte Signatures***

We explored the correlation between the BFSigs and the signatures of monocyte-associated venetoclax resistance, developed by White. et al ^11^. The monocyte signatures were calculated by compressing the expression of BCL3, CD14, LILRB1, LRP1, MAFB, PSAP, SLC15A3, and SLC7A7 using GSVA R package ^12^. We analyzed the correlation between BFSigs and the monocye signatures using Spearman's method.

***French-American-British (FAB) Classification***

We analyzed the relationship between the BFSigs and French-American-British (FAB) classification of AML. For the BeatAML dataset, clinical data were acquired from the original study ^1^. Clinical data of LeuceGene was publicly available at https://data.leucegene.iric.ca. We downloaded FAB information of TCGA_LAML dataset at https://figshare.com/s/7c683384c6e2add08262. We compared expression of the BFSigs between FAB classes using Wilcoxon rank-sum test.

***Patient samples Preparation***

Human bone marrow mononuclear cells (MNCs) from patients with AML were isolated using Ficoll gradient method and cryopreserved in Cell banker 2, a serum-free medium. The MNCs of the patients were thawed at 37℃ in Iscove’s modified Dulbecco’s media containing 10% fetal bovine serum before the experiment.

***Apoptosis Assay***

The ability of venetoclax to induce apoptosis was measured using Dead Cell Apoptosis Kit with Annexin V FITC and PI (Thermo Scientific) according to the manufacturer’s instructions. Primary cells (5 x 105/a well ~ 1 x 106/a well) in 6-well plates were treated with venetoclax from 0 to 10 µM for 24 h. Apoptotic cells were analyzed using BD FACSymphony A3 (BD Biosciences). Cells stained with Annexin V were classified as early apoptotic cells, and those stained with Annexin V and PI were late apoptotic cells. The results were analyzed using FlowJo 10.7.1 software (Tree Star, Ashland, OR, USA). Apoptosis induction rate was calculated as the difference between apoptosis rate at 0 µM and 10 µM.

**References**

1 Tyner, J. W. et al. Functional genomic landscape of acute myeloid leukaemia. Nature 562, 526-531 (2018).

2 Bisaillon, R. et al. Genetic characterization of ABT-199 sensitivity in human AML. Leukemia 34, 63-74 (2020).

3 Mammoliti, A. et al. Orchestrating and sharing large multimodal data for transparent and reproducible research. Nature communications 12, 1-10 (2021).

4 Liao, Y., Smyth, G. K. & Shi, W. The Subread aligner: fast, accurate and scalable read mapping by seed-and-vote. Nucleic acids research 41, e108-e108 (2013).

5 Liao, Y., Smyth, G. K. & Shi, W. featureCounts: an efficient general purpose program for assigning sequence reads to genomic features. Bioinformatics 30, 923-930 (2014).

6 Hansen, K. D., Irizarry, R. A. & Wu, Z. Removing technical variability in RNA-seq data using conditional quantile normalization. Biostatistics 13, 204-216 (2012).

7 Subramanian, A. et al. Gene set enrichment analysis: a knowledge-based approach for interpreting genome-wide expression profiles. Proceedings of the National Academy of Sciences 102, 15545-15550 (2005).

8 Lee, C. cslee159/OptimalGeneNMF(v1.0.0). Github. 10.5281/zenodo.6901509 (2022).

9 Lin, X. & Boutros, P. C. Fast Nonnegative Matrix Factorization and Applications to Pattern Extraction, Deconvolution and Imputation. BioRxiv, 321802 (2018).

10 Smyth, G. K., Ritchie, M., Thorne, N. & Wettenhall, J. LIMMA: linear models for microarray data. In Bioinformatics and Computational Biology Solutions Using R and Bioconductor. Statistics for Biology and Health. (2005).

11 White, B. S. et al. Bayesian multi-source regression and monocyte-associated gene expression predict BCL-2 inhibitor resistance in acute myeloid leukemia. NPJ precision oncology 5, 1-11 (2021).

12 Hänzelmann, S., Castelo, R. & Guinney, J. GSVA: gene set variation analysis for microarray and RNA-seq data. BMC bioinformatics 14, 1-15 (2013).
