# Supplementary material for: Transcriptional signatures of the BCL2 family for individualized acute myeloid leukaemia treatment
Source: Genome Med. 2022 Sep 28;14:111. doi: 10.1186/s13073-022-01115-w (PMC9520894; doi:10.1186/s13073-022-01115-w)
Supplement: Supplementary file 2 — Additional file 2: Fig S1. Gene optimization. Fig S2. Histogram of venetoclax IC50 values from the BeatAML dataset. Fig S3. Optimal rank selection for NMF. Fig S4. Association between the BCL2 family and venetoclax response. Fig S5. Contribution of the BCL2 family to signatures. Fig S6. Identification of BCL2 family-based subtypes in other hematologic malignancies. Fig S7. Mutation status of BCL2 family-based subtypes. Fig S8. Comparison of BCL2 family-based acute myeloid leukaemia (AML) subtypes based on drug response. Fig S9. Assignment of BFSig subtypes in Tavor Dataset. Fig S10. Batch effect correction. Fig S11. External validation of BCL2 family signature-based classifier. Fig S12. Profile of BCL2 family signatures in acute myeloid leukaemia (AML) cell lines. Fig S13. Prediction response to BCL2 family inhibitors in cell lines. Fig S14. Improvement of Prediction Power via Gene Optimization and Rank Selection. Fig S15. Prediction Power using Optimized Genes in External Datasets. Fig S16. Venetoclax Response in NanoString Samples. Fig S17. Relationship between BFL1/MCL1 signature and FAB classification of AML. Fig S18. Correlation between BCL2 family signatures and monocyte signatures. Fig S19. Scheme of gene optimization algorithm. Fig S20. Performance Measurements in Imputation of BCL2 family Profiles. [file 13073_2022_1115_MOESM2_ESM.docx]

**Supplementary Figures**


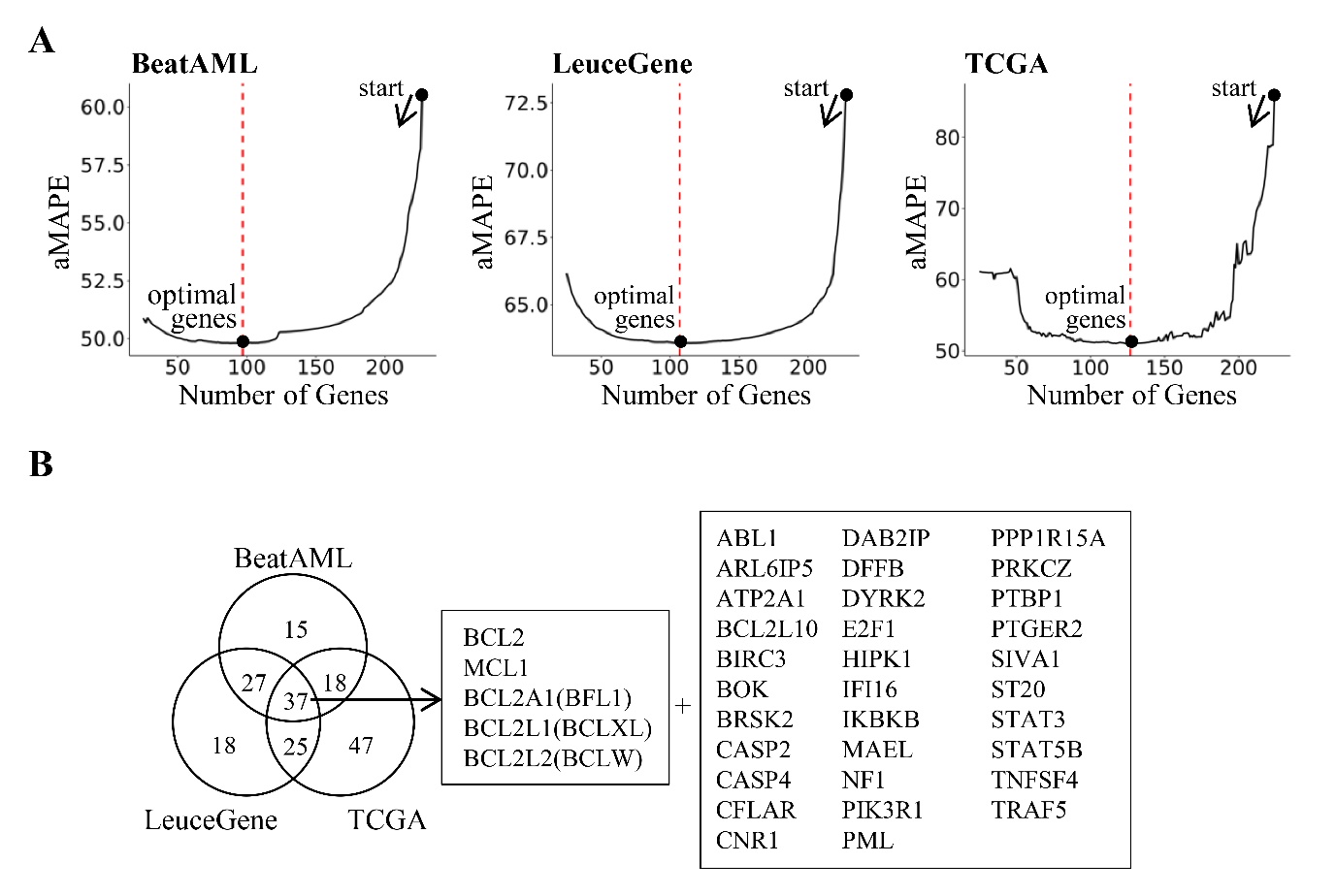


**Fig S1. Gene optimization**

(A) Decrease of recovery error of anti-apoptotic BCL2 family genes during gene optimization in each dataset. Genes with minimal recovery error are selected as optimized genes. aMAPE indicates an average of mean absolute percentage error. (B) Venn diagram illustrating the optimized genes in each of the three AML datasets. Thirty-seven intersecting genes are listed on the right of the figure. All NMF calculations were conducted using rank 3, which is the optimal rank in the three datasets.


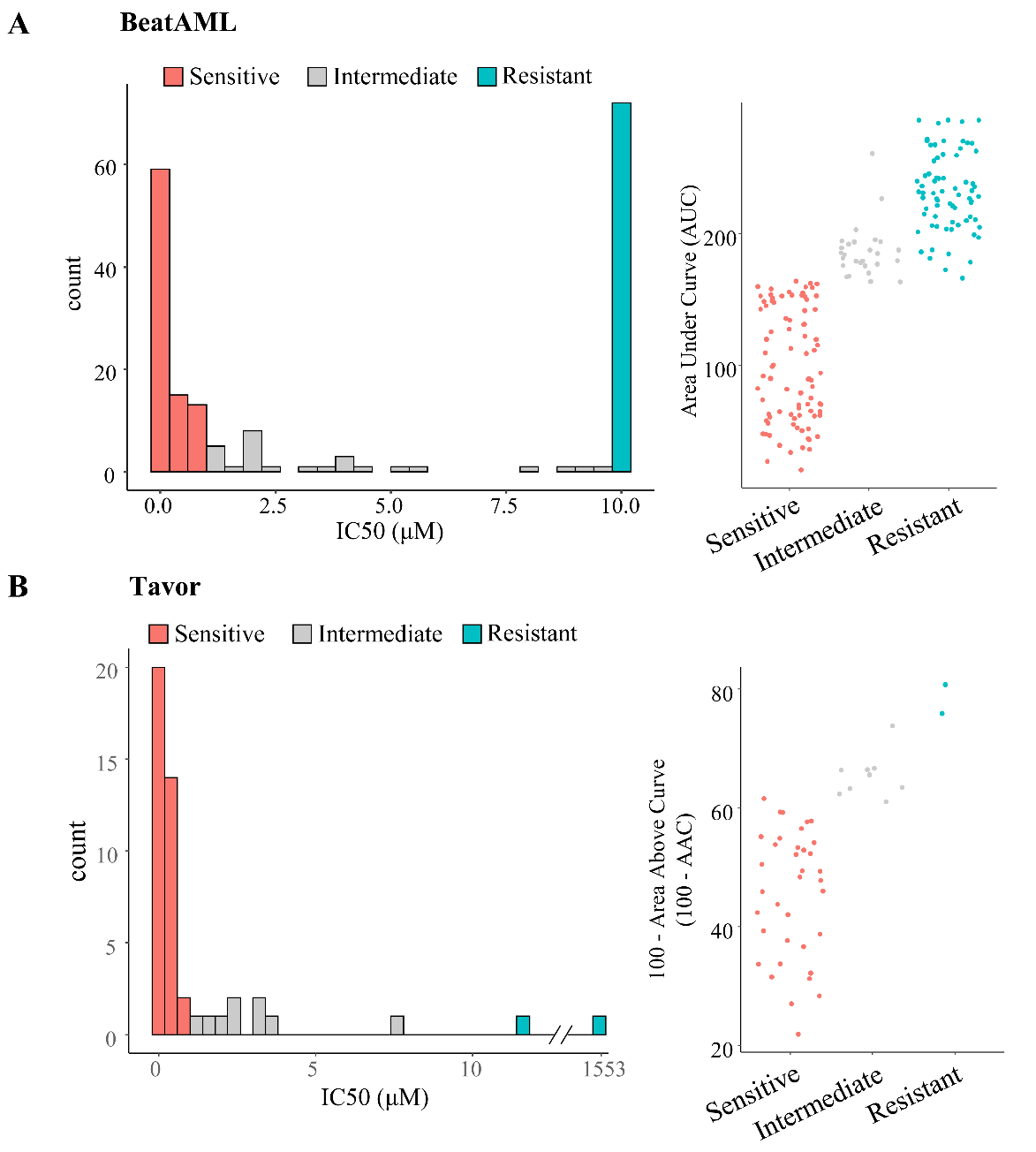


**Fig S2. Histogram of venetoclax IC50 values from the BeatAML dataset**

IC50 values are binarized to sensitive if IC50 ≤ 1 µM and resistant if IC50 ≥ 10 uM. (A) In BeatAML dataset, 87, 72, and 27 samples are allocated to the sensitive, resistant, and intermediate groups, respectively. (B) In Tavor dataset, 36, 2, and 9 samples are allocated to the sensitive, resistant, and intermediate groups, respectively. The right panels represent that the sensitive and resistant groups don’t overlap in AUC or AAC from drug curves.


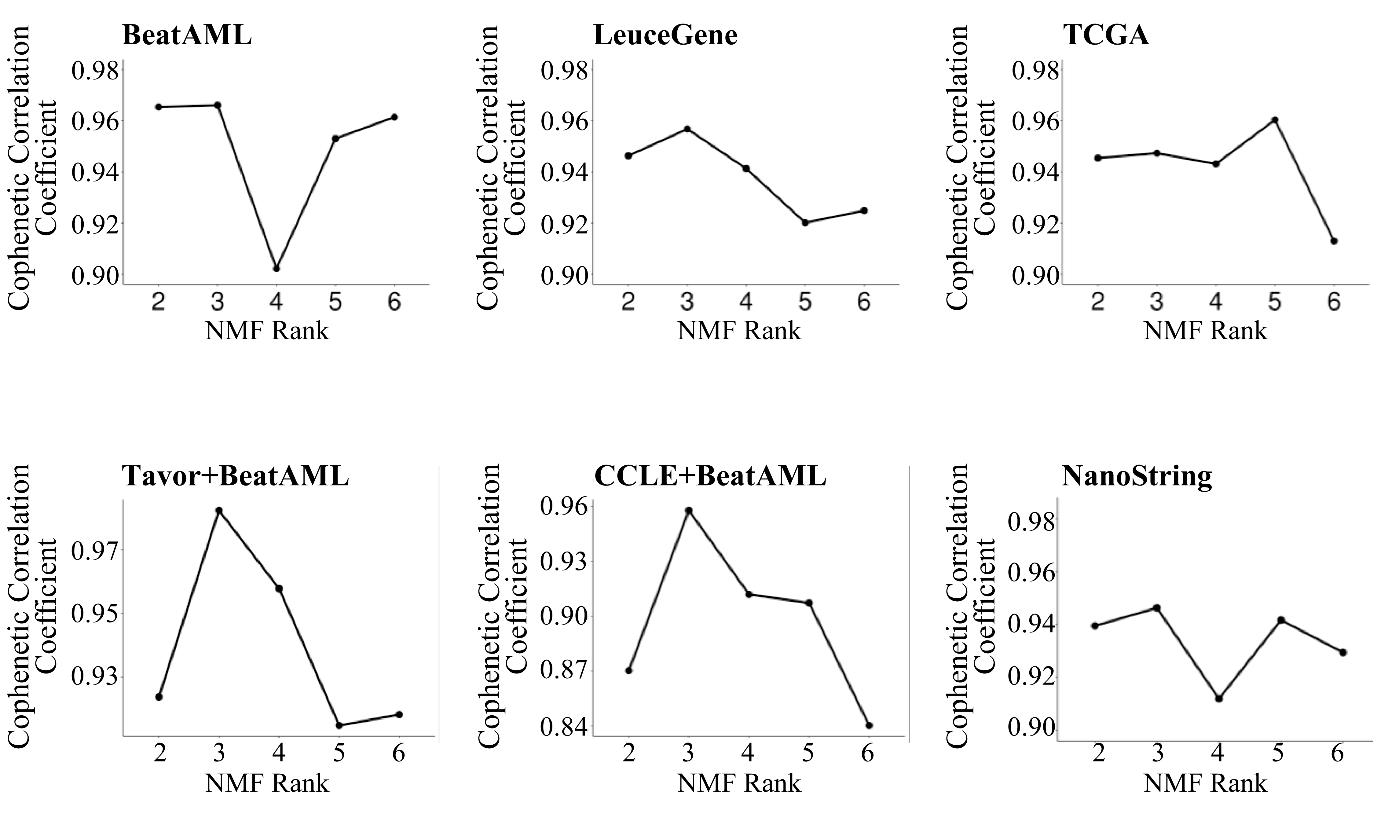


**Fig S3. Optimal rank selection for NMF**

Cophenetic correlation coefficients of a given rank and optimized genes. Rank 3 is the first rank in which the cophenetic correlation coefficient begins to fall in all AML datasets.


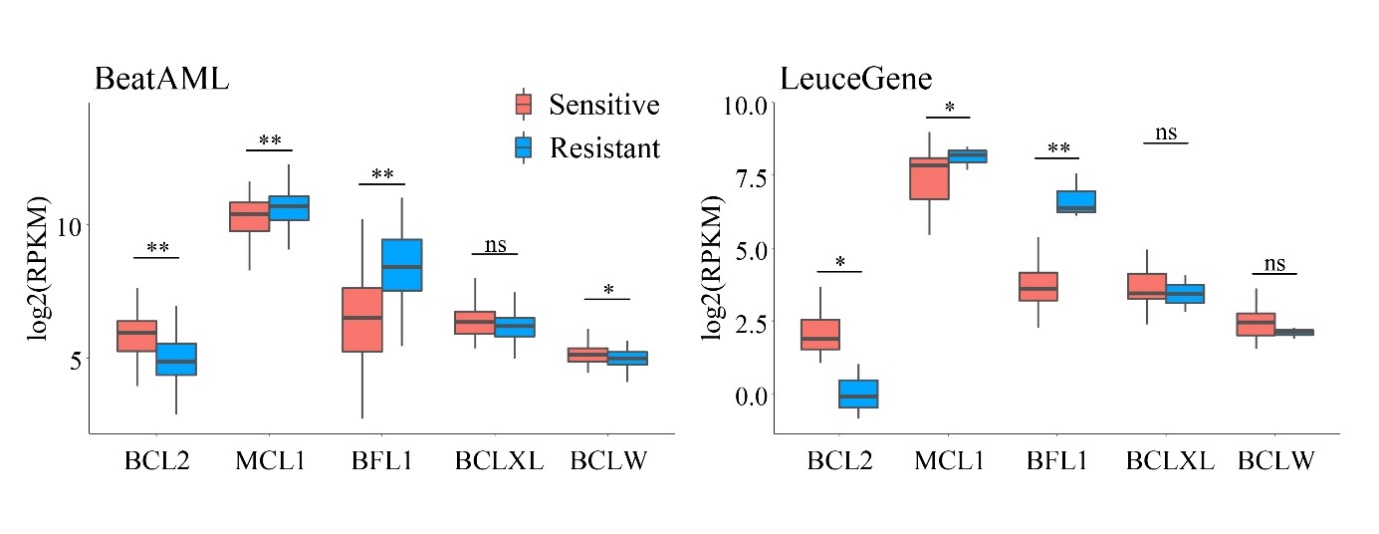


**Fig S4. Association between the BCL2 family and venetoclax response**

Comparison of expression of anti-apoptotic BCL2 family genes between venetoclax-sensitive and -resistant groups in BeatAML (81 sensitive and 72 resistant) and LeuceGene (20 sensitive and 3 resistant). Note that we only used a subset of LeuceGene in which publicly available response information. Differentially expressed BCL2 and BFL1 genes were already identified in the original LeuceGene study (Bisaillon R. et al.). P-values are calculated by Welch’s t-test. * <0 .05, ** < 0.01, ns > 0.10.


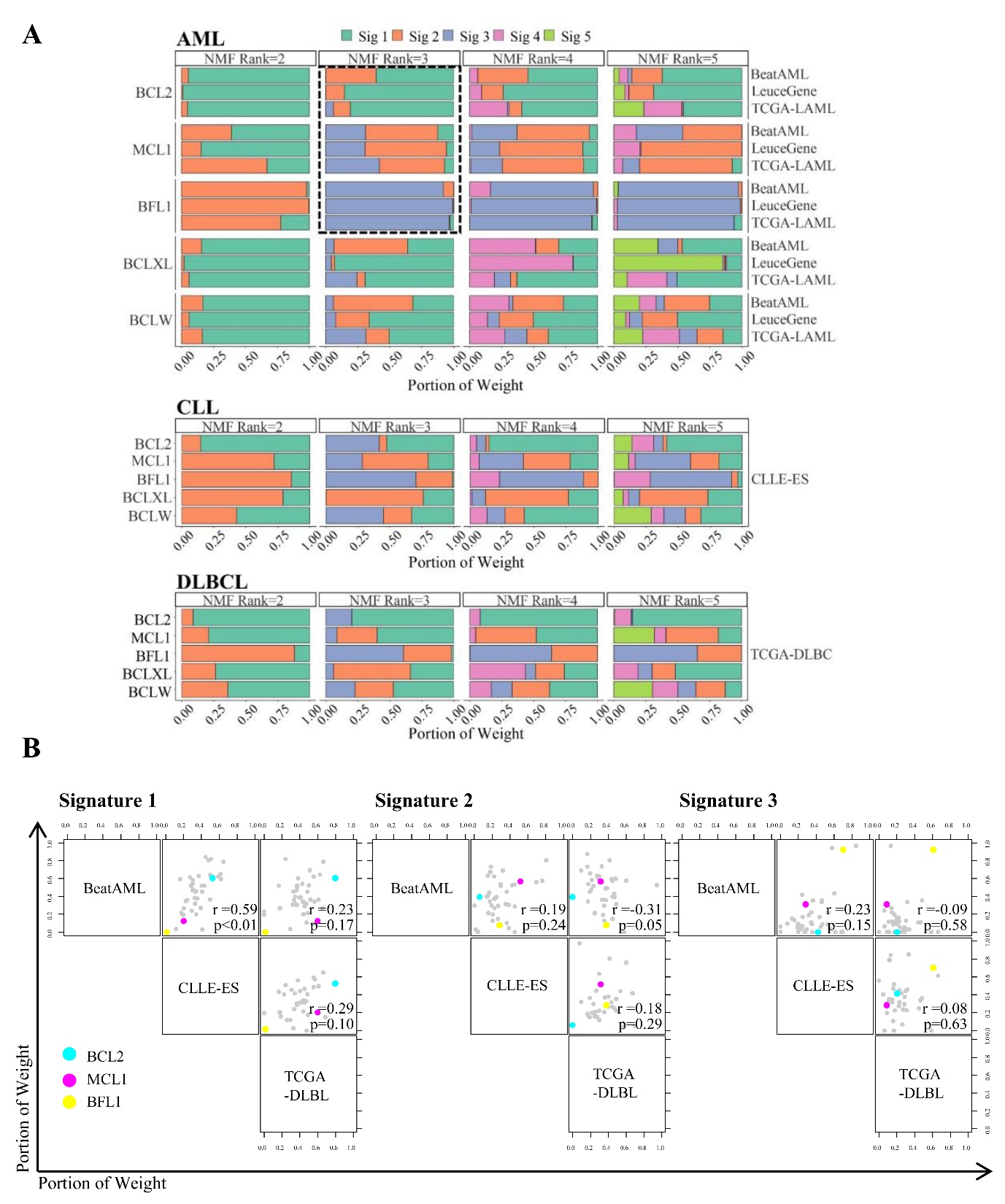


**Fig S5. Contribution of the BCL2 family to signatures**

(A) Weight of the anti-apoptotic BCL2 family in defining the BCL2 family signatures (BFSigs) in hematologic malignancies (AML, CLL, and DLCBL). In AML, the dotted box indicates outcomes used to interpret signatures in this study; signatures 1, 2, and 3 indicate BCL2, MCL1/BCL2, and BFL1/MCL1 signatures, respectively (refer to Figure 3 and 4). The signatures were originally extracted in a random order, thus we manually reordered them to be similar to other ranks’ results. The signatures were manually reordered for the other malignancies to be similar to the AML results. Each weight is normalized to sum 1. (B) Correlation plot of the weight of the optimized genes between hematologic malignancies. Three types of hematologic malignancies show the inconsistent weight of optimized genes (*p* > 0.05) except signature 1 (BCL2 signature) of CLLE-ES (*p* < 0.01). Each weight of genes is normalized to sum 1. Correlation coefficients are calculated using Spearman’s rho. (AML: acute myeloid leukaemia, CLL: chronic lymphocytic leukaemia, DLBCL: diffuse large B-cell lymphoma)


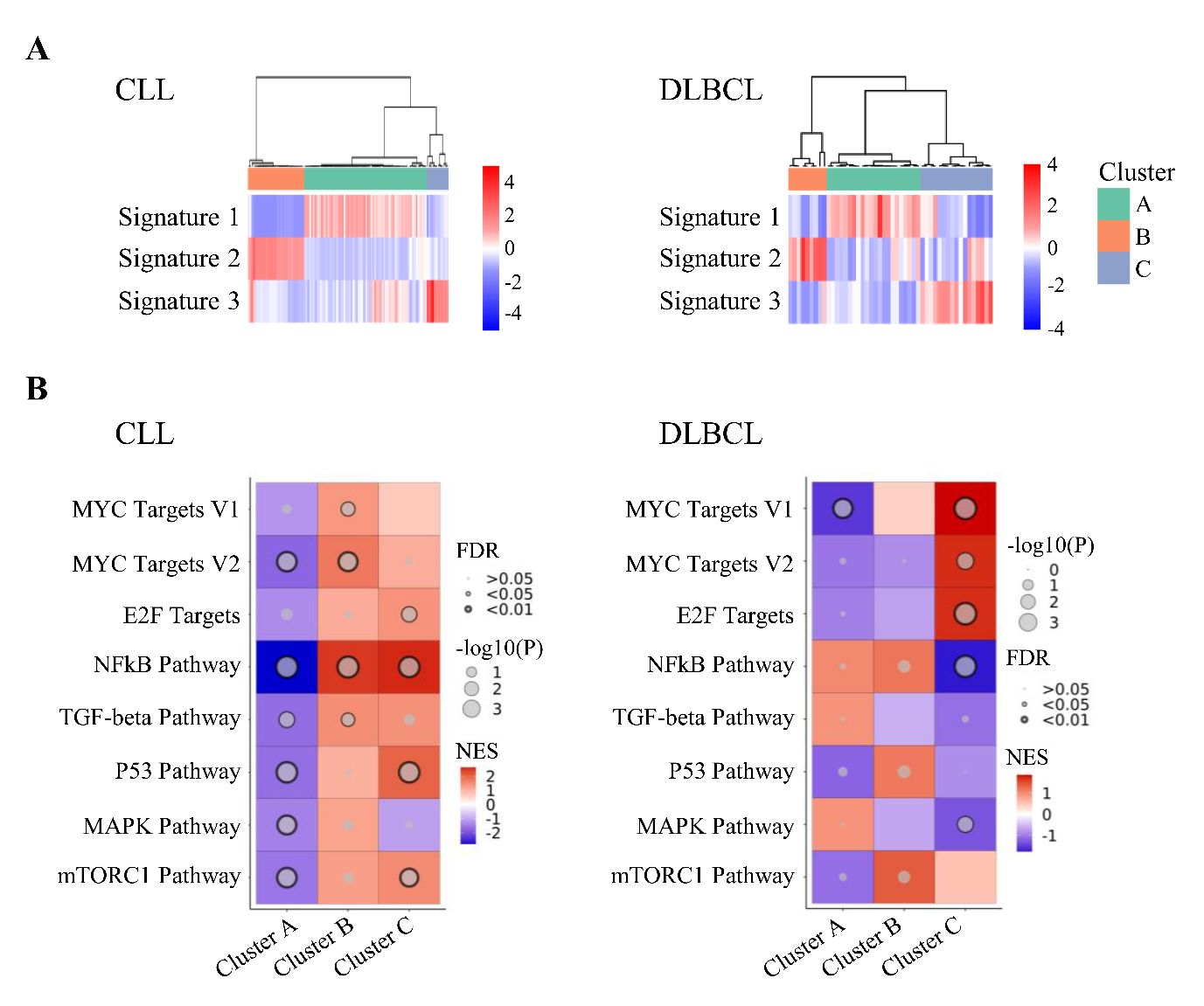


**Fig S6. Identification of BCL2 family-based subtypes in other hematologic malignancies**

(A) Profile of BCL2 family transcriptional signatures in other hematologic malignancies (CLL and DLBCL). Weights of the BCL2 family in signatures are described in Fig S5 (NMF Rank=3). Clustering is conducted after row scaling. Cluster A, B, and C indicate signature 1, 2, and 3-enriched samples. (B) Gene Set Enrichment Analysis (GSEA) from comparing one cluster and the others identifies enriched gene sets in each cluster. NES indicates a normalized enrichment score. The source of the gene sets is the same as in Figure 5. AML: acute myeloid leukaemia, CLL: chronic lymphocytic leukaemia, DLBCL: diffuse large B-cell lymphoma.


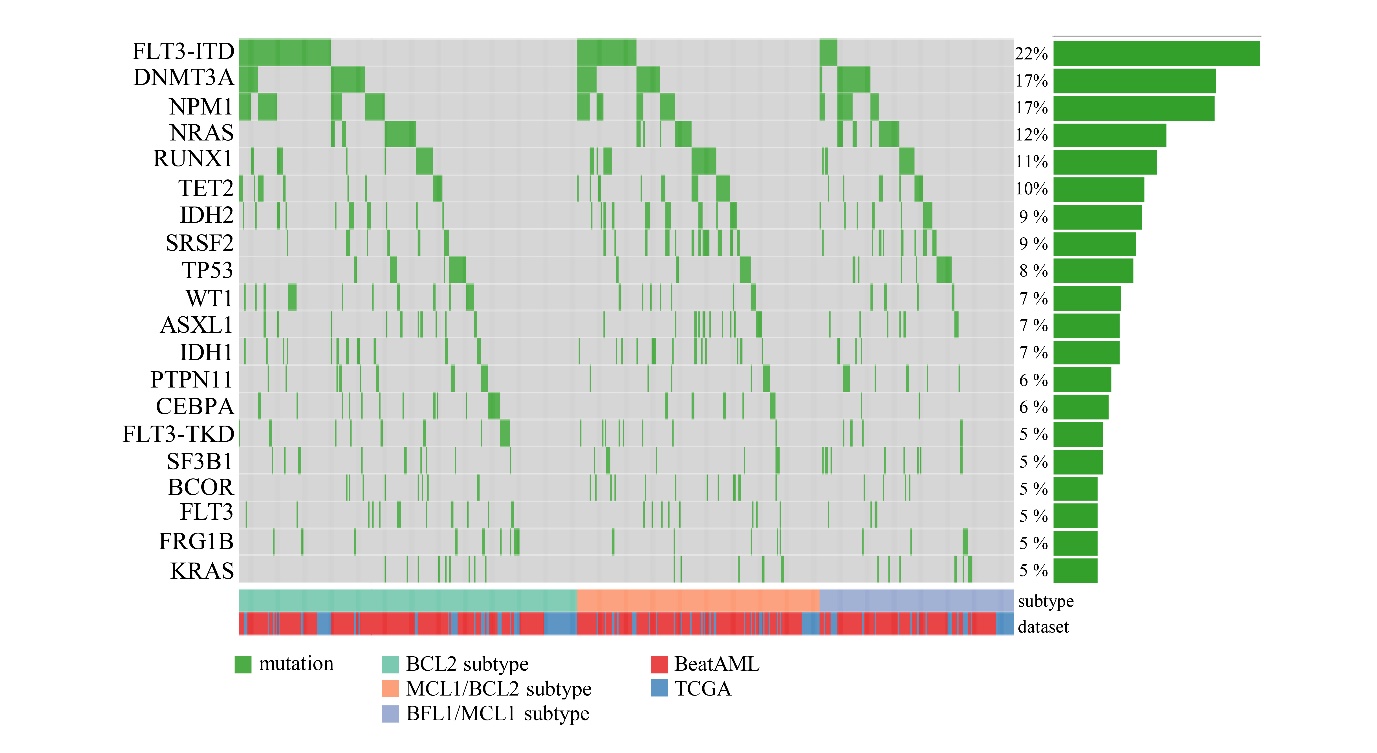


**Fig S7. Mutation status of BCL2 family-based subtypes**

In oncoplot, protein-changing mutations of each BCL2 family-based subtype are represented. The top 20 genes are shown. The mutation prevalence of each gene was not significantly different between BFSig subtypes (chi-square test *p* = 1 except SF3B1 with *p* = 0.33). The first bar indicates BCL2, MCL1/BCL2, and BFL1/MCL1 subtypes and the second bar shows the patient cohort (BeatAML and TCGA).


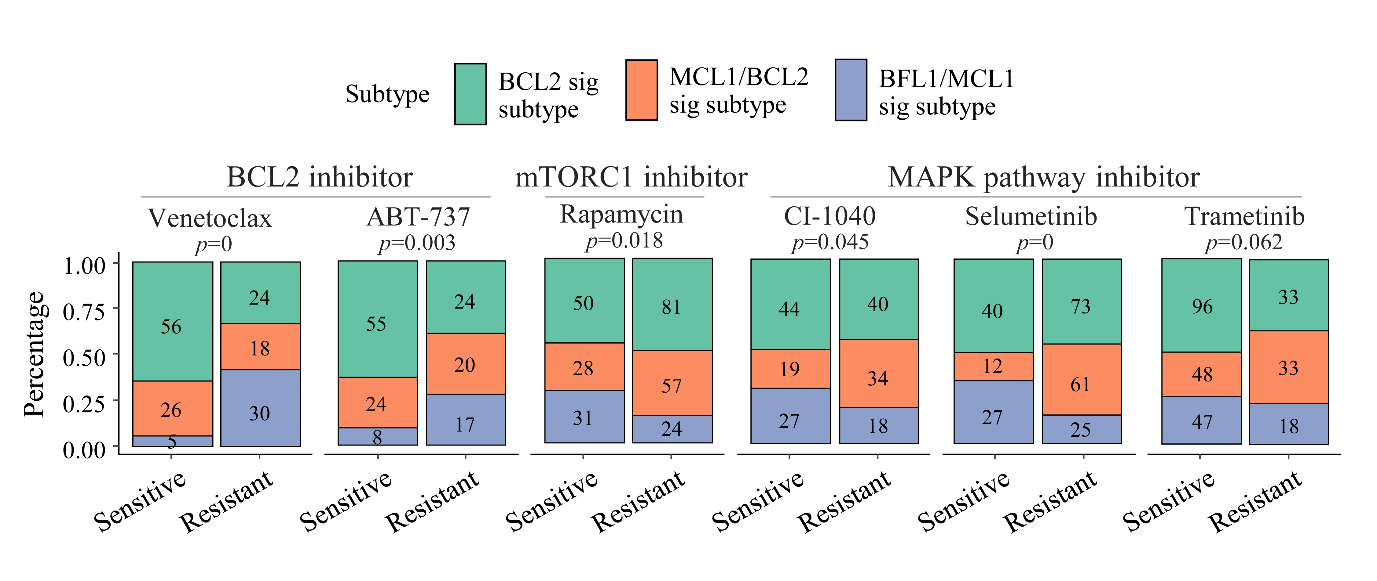


**Fig S8. Comparison of BCL2 family-based acute myeloid leukaemia (AML) subtypes based on drug response**

The proportion of BFSig-based subtypes by response groups in BeatAML dataset. Numbers in bars indicate the number of samples included in given subtypes. The drugs are sorted by the proportion of BFL1/MCL1 subtype. P-values are calculated using the chi-square test to compare the number of samples included in the subtypes. The response groups were appointed by binarizing IC50 values to be sensitive if IC50 ≤ 1µM and resistant if IC50 ≥ 10µM. IC50 data was provided from the original BeatAML study (Tyner, J. W. et al.).


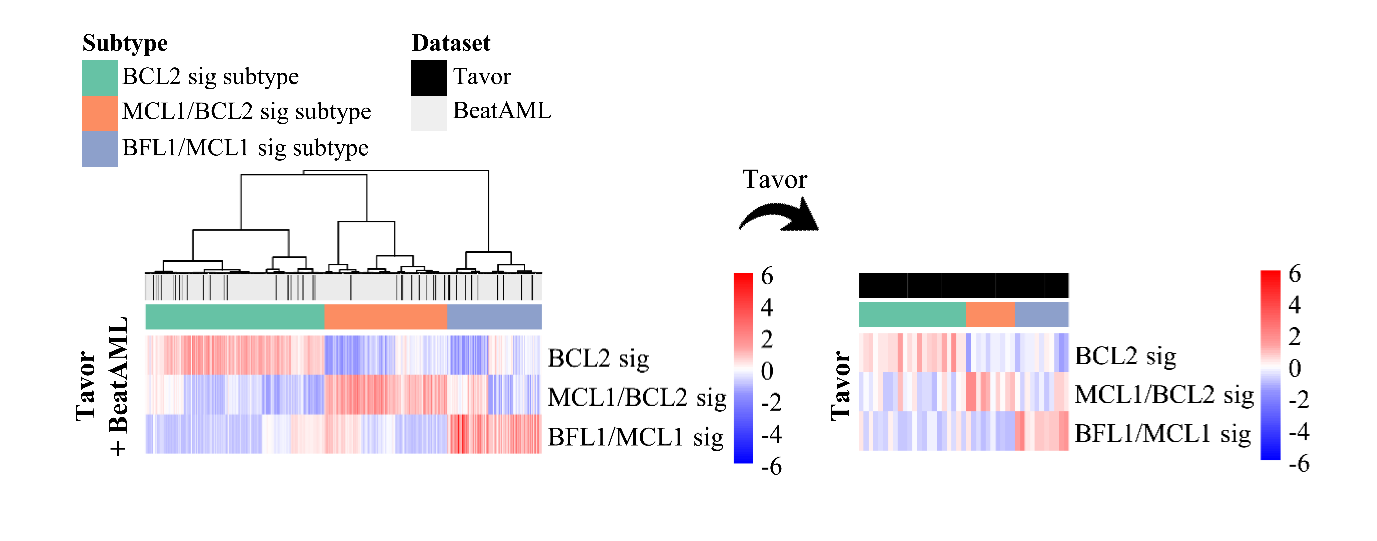


**Fig S9. Assignment of BFSig subtypes in Tavor Dataset**

Tavor dataset is combined with BeatAML to overcome an insufficient number of samples to conduct clustering. The dataset shows the three distinct clusters annotated as BCL2, MCL1/BCL2, and BFL1/MCL1 signature subtypes. Columns are clustered using hierarchical clustering with average distance.

**
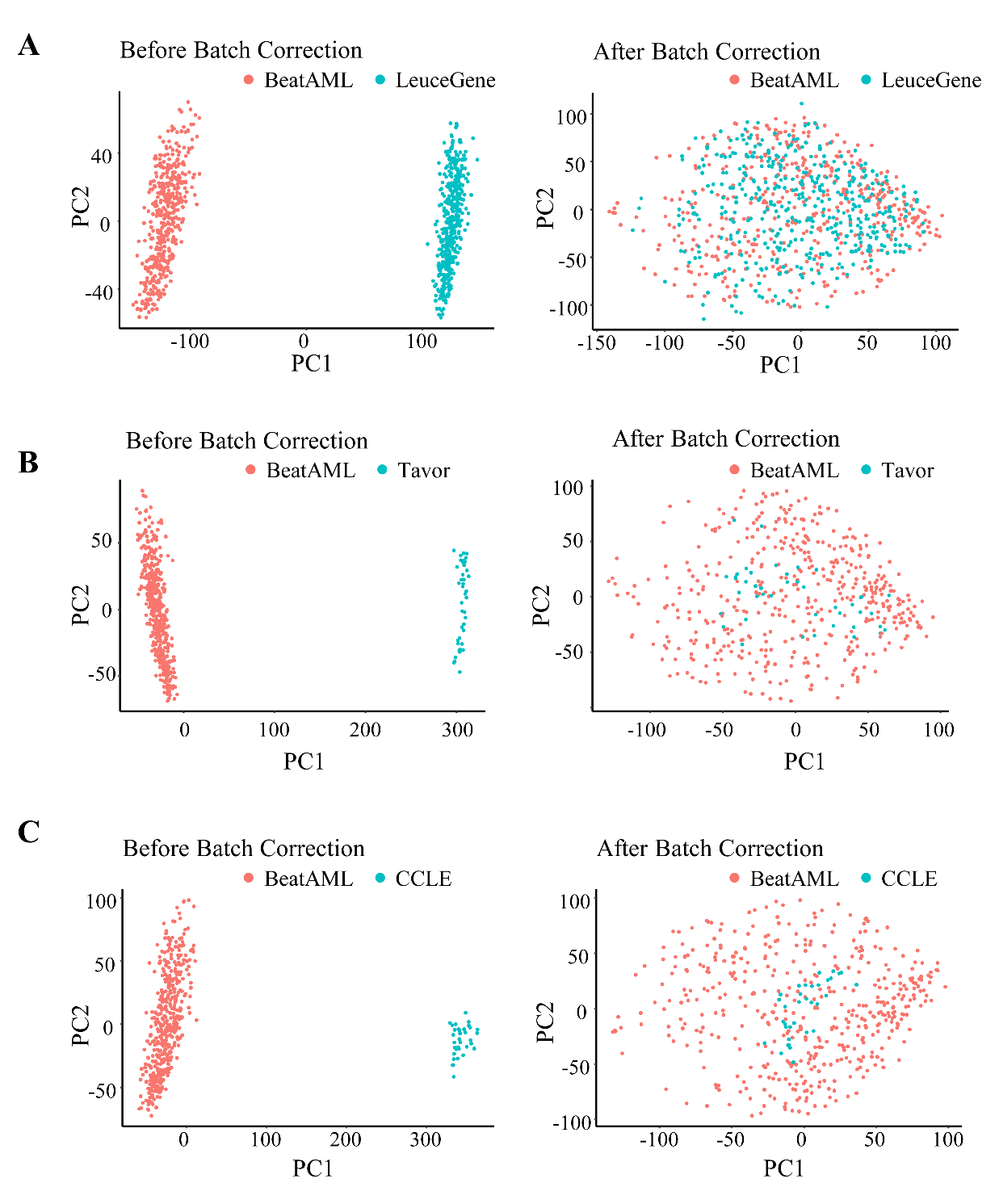
**

**Fig S10. Batch effect correction**

Principal component analysis (PCA) from expression profiles before and after batch correction between (A) BeatAML and LeuceGene, (B) BeatAML and Tavor, and (C) BeatAML and CCLE.

**
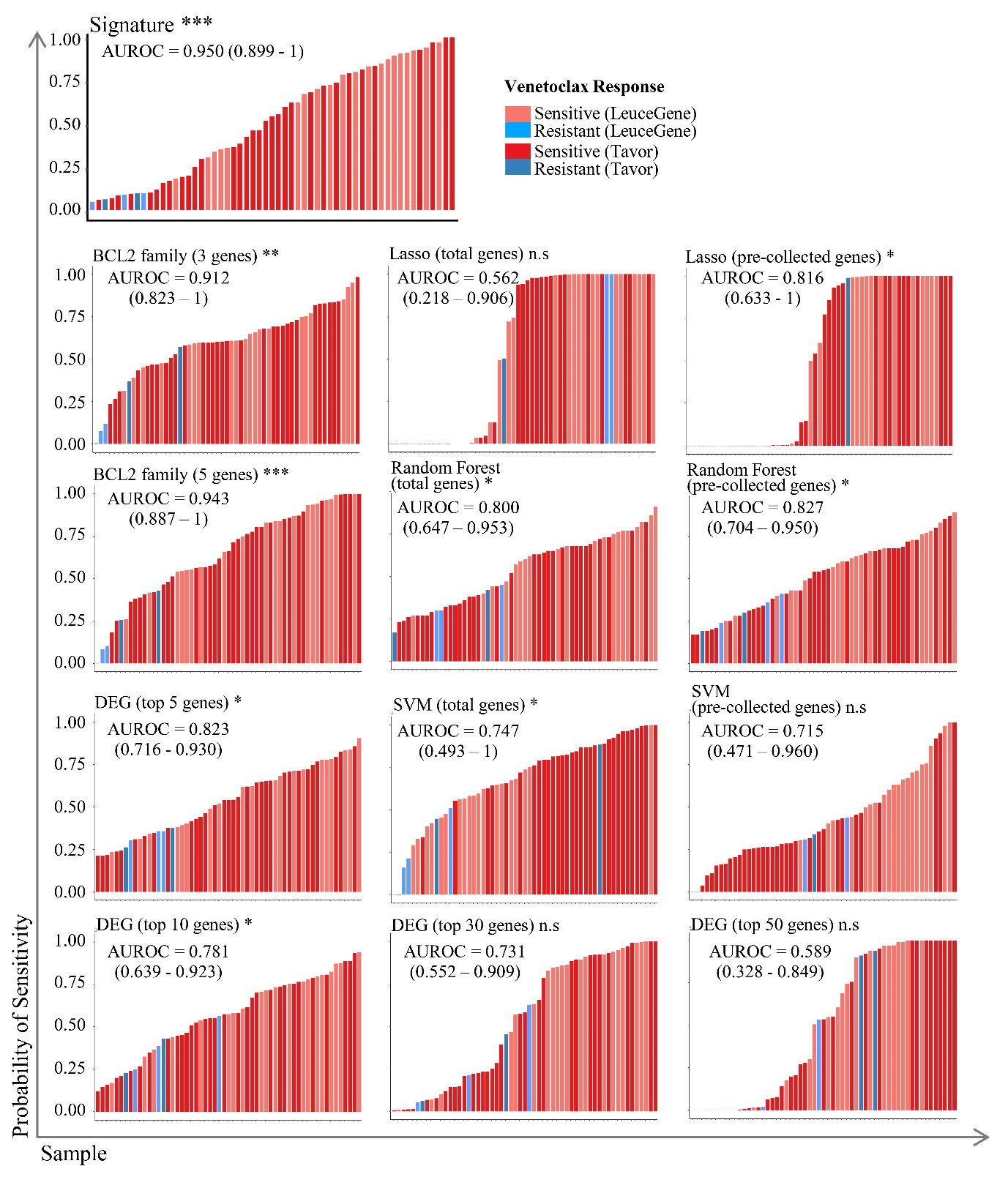
**

**Fig S11. External validation of BCL2 family signature-based classifier**

Comparison of prediction performance between venetoclax response classifiers in LeuceGene and Tavor (external validation sets). Only samples assigned to the sensitive or resistant groups are represented. Bar graphs show an estimate of the probability (y hat) of individuals. Used variables and models are the same as in Figure 6. The numbers below of the AUROC represent 95% confidence interval. P-values next to each title are calculated using Wilcoxon rank-sum test by comparing the probability rank between the response groups. * < 0.05, ** < 0.01, *** < 0.001, ns > 0.05


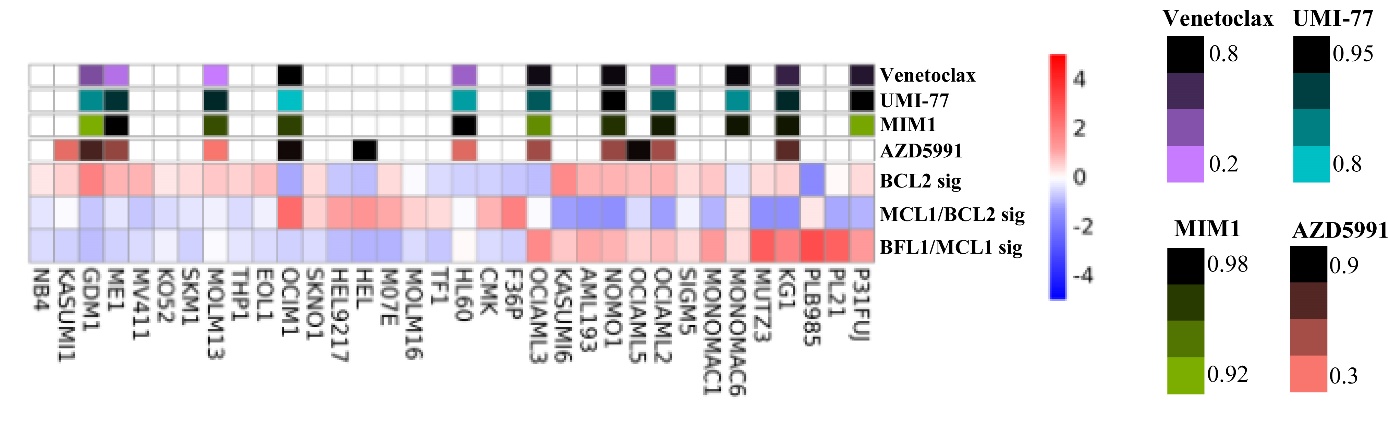


**Fig S12. Profile of BCL2 family signatures in acute myeloid leukaemia (AML) cell lines**

Heatmap of BFSig of AML cell lines from CCLE dataset. Venetoclax and MCL1 inhibitor (MIM1, UMI-77, and AZD5991) AUC (area under the dose-response curve) information is from GDSC2. Clustering is conducted after row scaling.


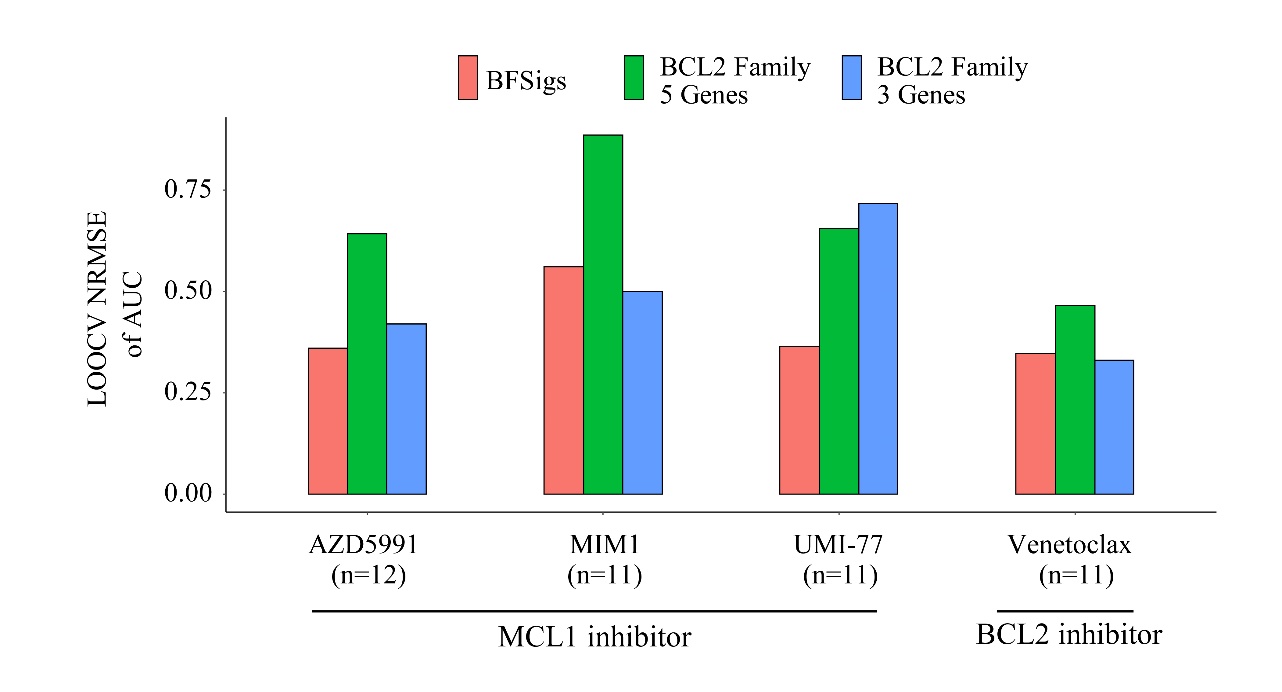


**Fig S13. Prediction response to BCL2 family inhibitors in cell lines**

Comparison of prediction performance between BFSig-based and original expression-based linear regression models for response to venetoclax or MCL1 inhibitors. Performance is measured using LOOCV NRMSE (Leave-One-Out Cross-Validation Normalized Root Mean Square Error) in predicting inhibitor response (AUC; area under the dose-response curve). NRMSE is calculated by RMSE / (max-min).


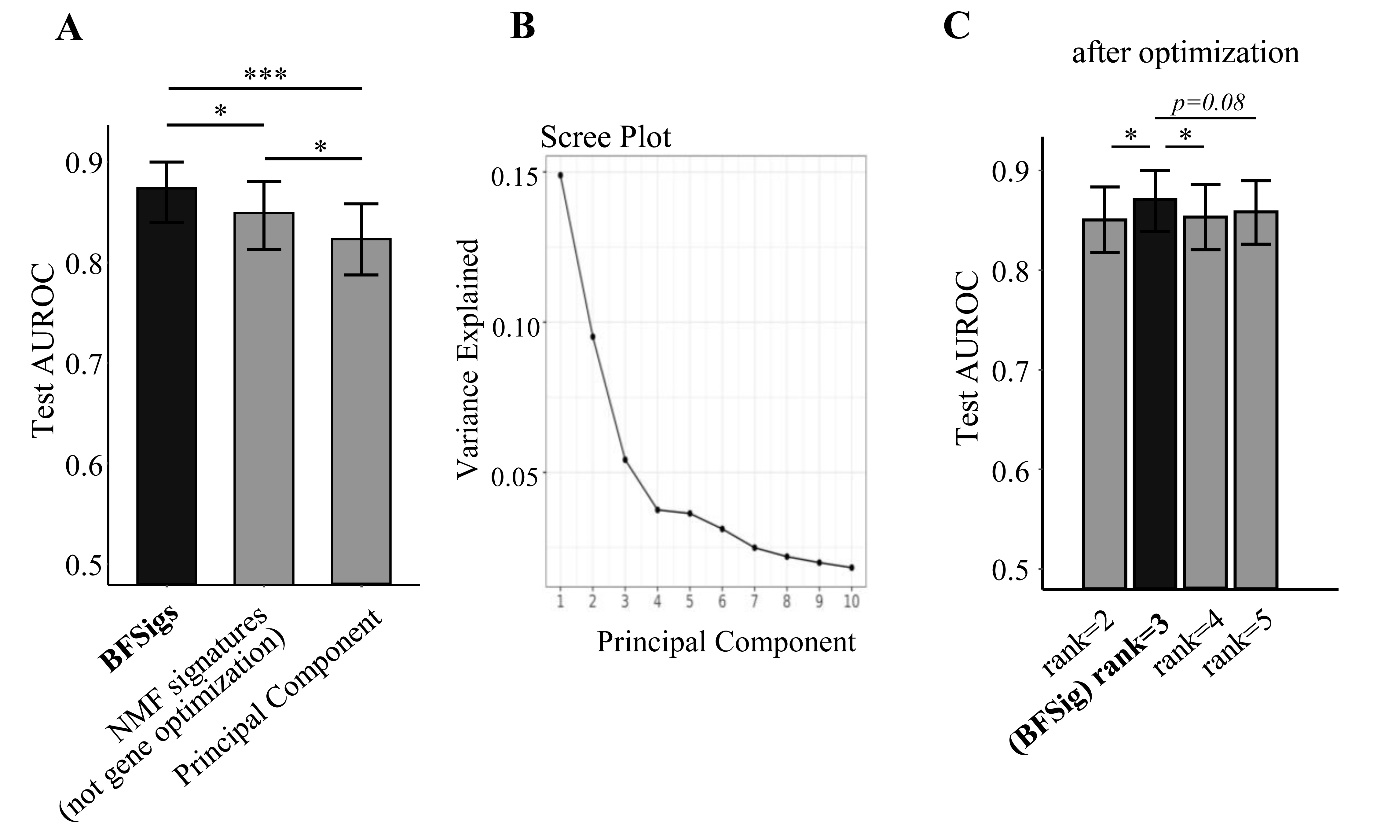


**Fig S14. Improvement of Prediction Power via Gene Optimization and Rank Selection**

Comparison of prediction performance between venetoclax response classifiers. (A) In the classification, the BFSigs calculated via gene optimization and the rank selection (rank=3) outperforms rank 3-based NMF signatures (without gene optimization) and three principal components calculated using pre-collected genes. (B) Scree plot of the principal components. (C) The BFSigs based on rank 3, selected via cophenetic correlation, outperforms other ranks-based signatures. Error bar indicates 95% confidence interval (CI). P values are calculated using the DeLong’s test. * < 0.05, ** < 0.01, *** < 0.001.


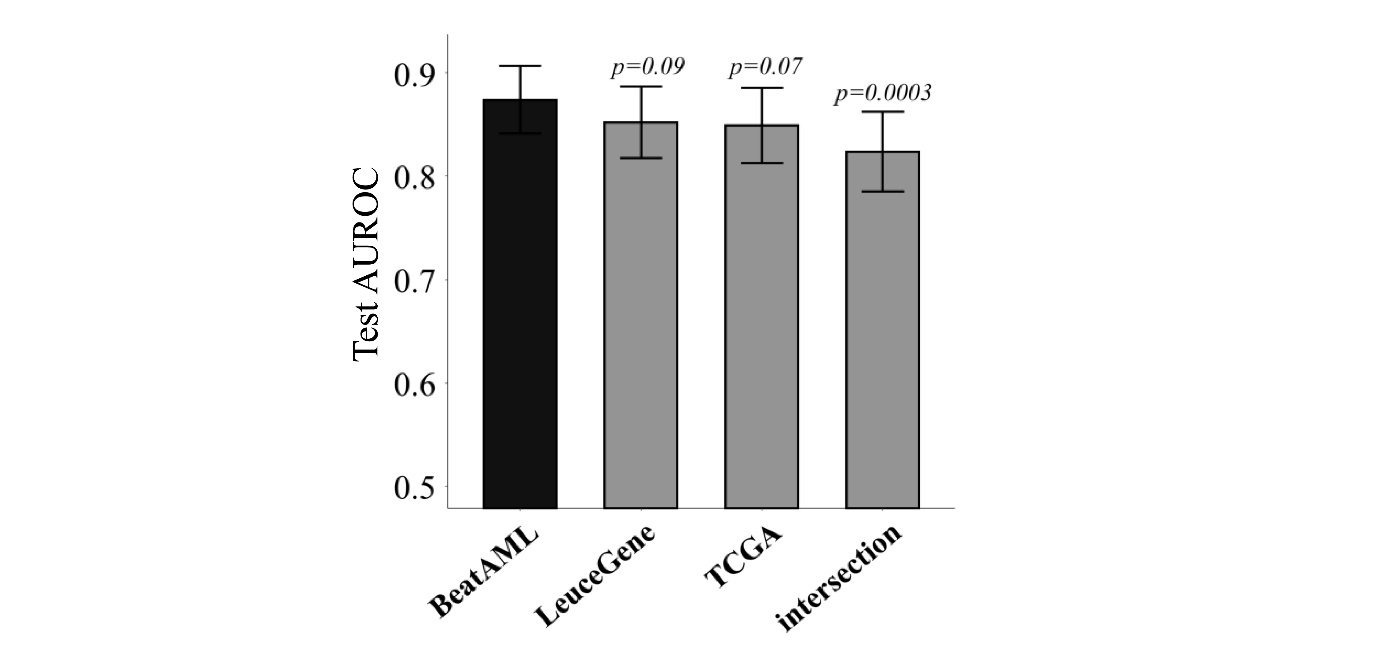


**Fig S15. Prediction Power using Optimized Genes in External Datasets**

In BeatAML classifier, a comparison of prediction performances between the signatures calculated using internally optimized genes, externally optimized genes (in LeuceGene or TCGA), and their intersection genes. Each optimized gene set are represented in Table S2. P values are calculated compared with BeatAML’s optimized genes using the DeLong’s test.


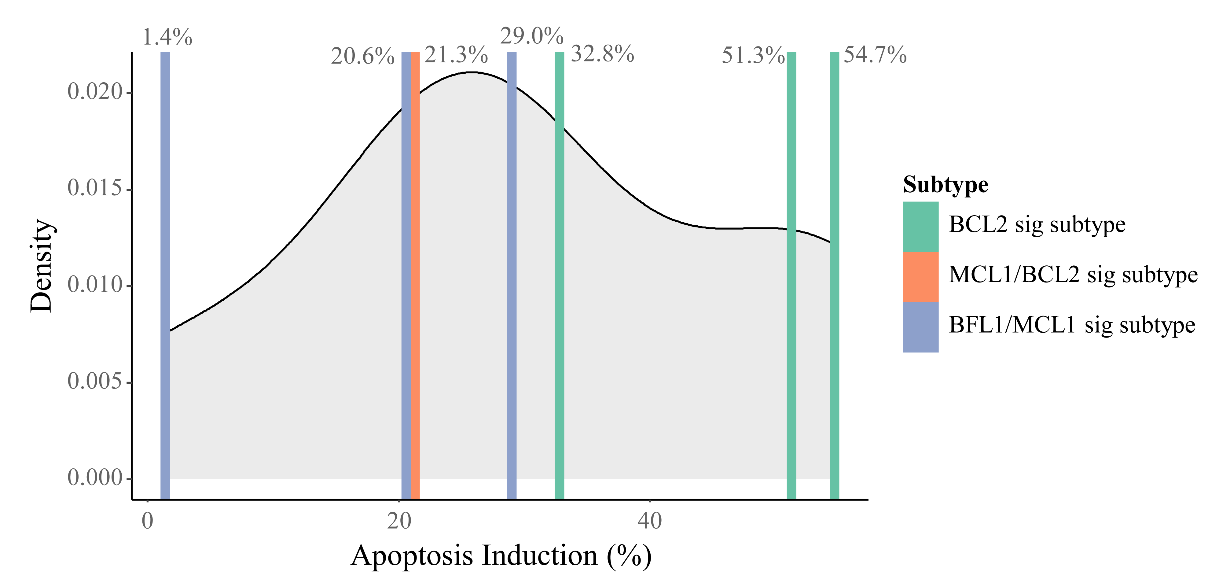


**Fig S16. Venetoclax Response in NanoString Samples**

Venetoclax-induced apoptosis induction rate in 7 samples sequenced using NanoString. The experiment is described in Supplementary Methods (Additional file 1). The distribution of the rate of 7 samples is represented in the background. Three samples assigned to the BCL2 signature subtype show higher apoptosis induction than samples in other subtypes.


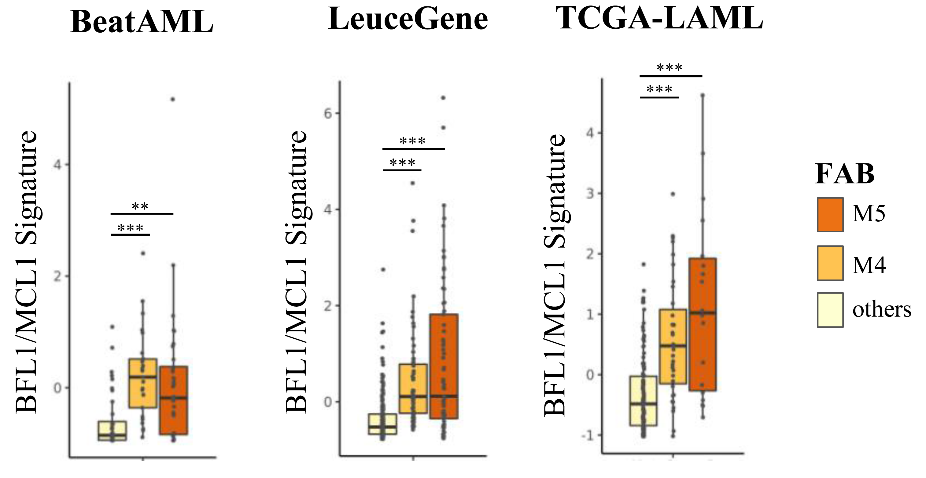


**Fig S17. Relationship between BFL1/MCL1 signature and FAB classification of AML**

Comparison of expression of BFL1/MCL1 signature between French-American-British (FAB) classes of AML. M4 and M5 represent acute myelomonocytic leukemia and acute monocytic leukemia, respectively. The signature on the y axis is normalized. P-values are calculated Wilcoxon rank-sum test. * < 0.05, ** < 0.01, ***<0.001, ns > 0.05.


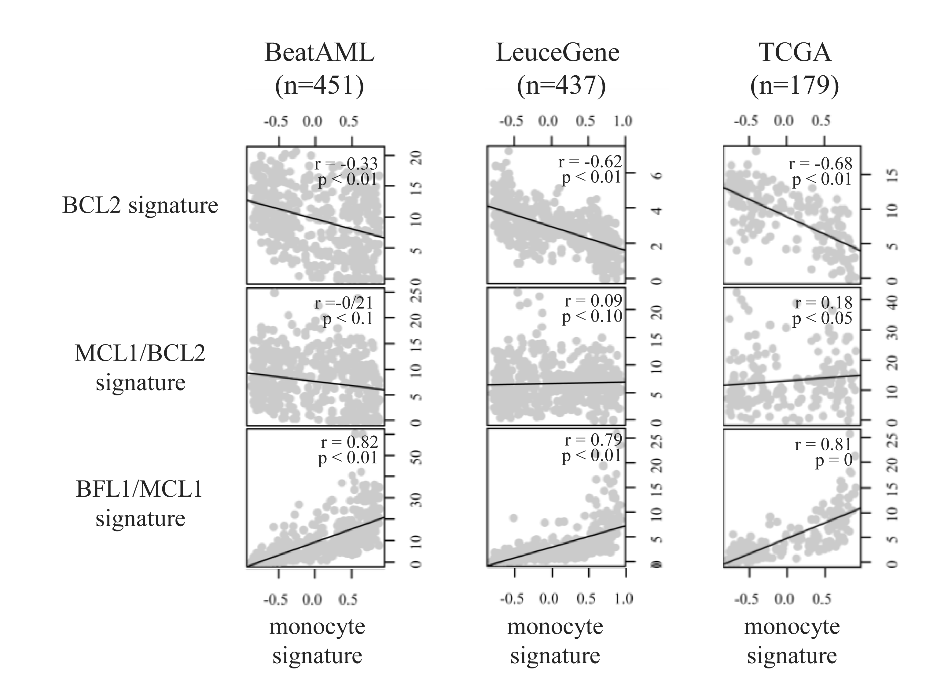


**Fig S18. Correlation between BCL2 family signatures and monocyte signatures**

Correlation analysis using the BCL2 family signatures (BFSigs) and the monocyte signatures. Especially, BFL1/MCL1 signature is significantly positively correlated with the monocyte signature. Correlation coefficients are calculated using Spearman’s rho.


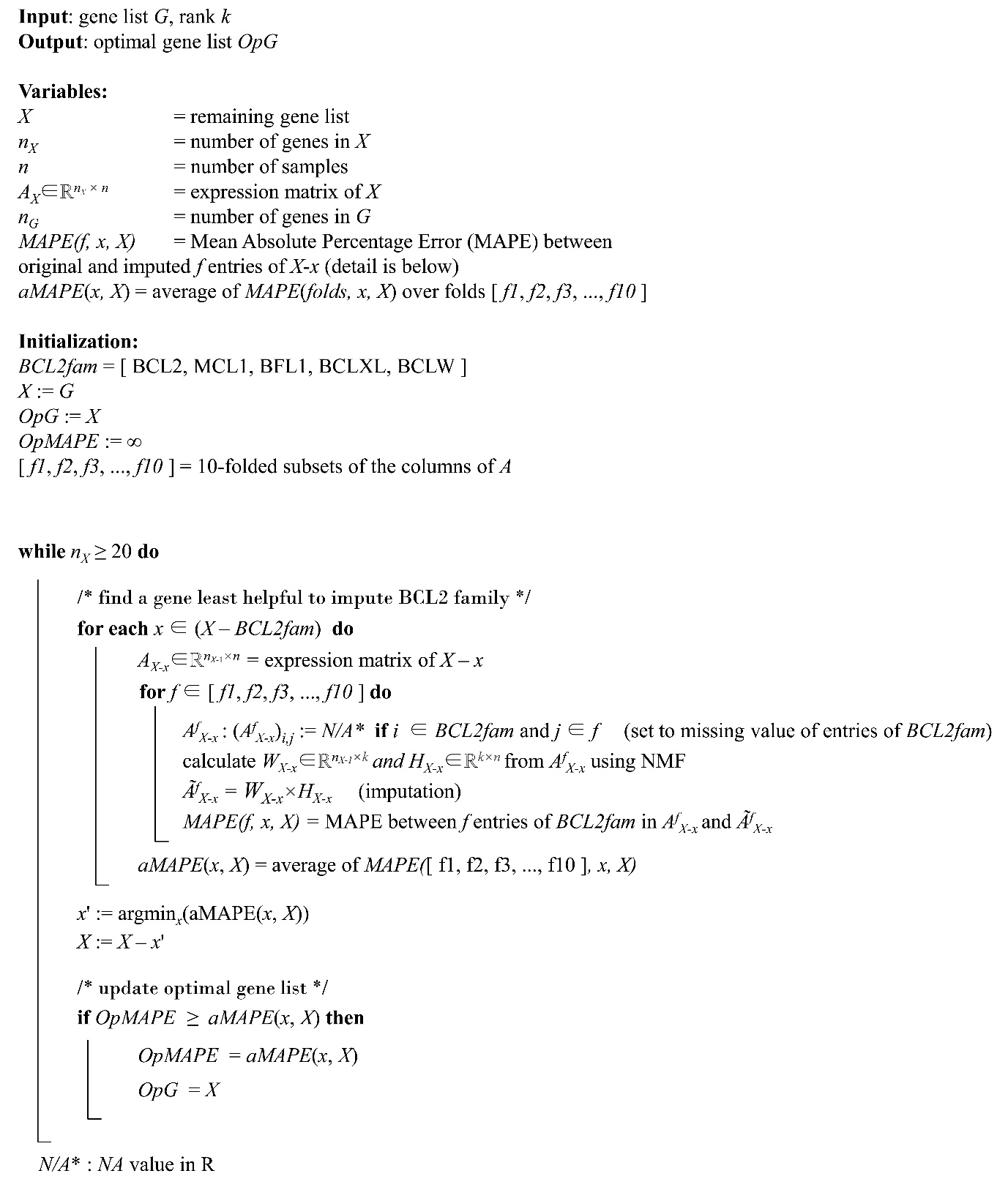


**Fig S19. Scheme of gene optimization algorithm**

Pseudocode described gene optimization to filter out noise genes not related to BCL2 family expression. For each round, a gene least helpful to impute expression of the anti-apoptotic BCL2 family is removed from pre-collected genes. The remaining genes are termed optimized genes.


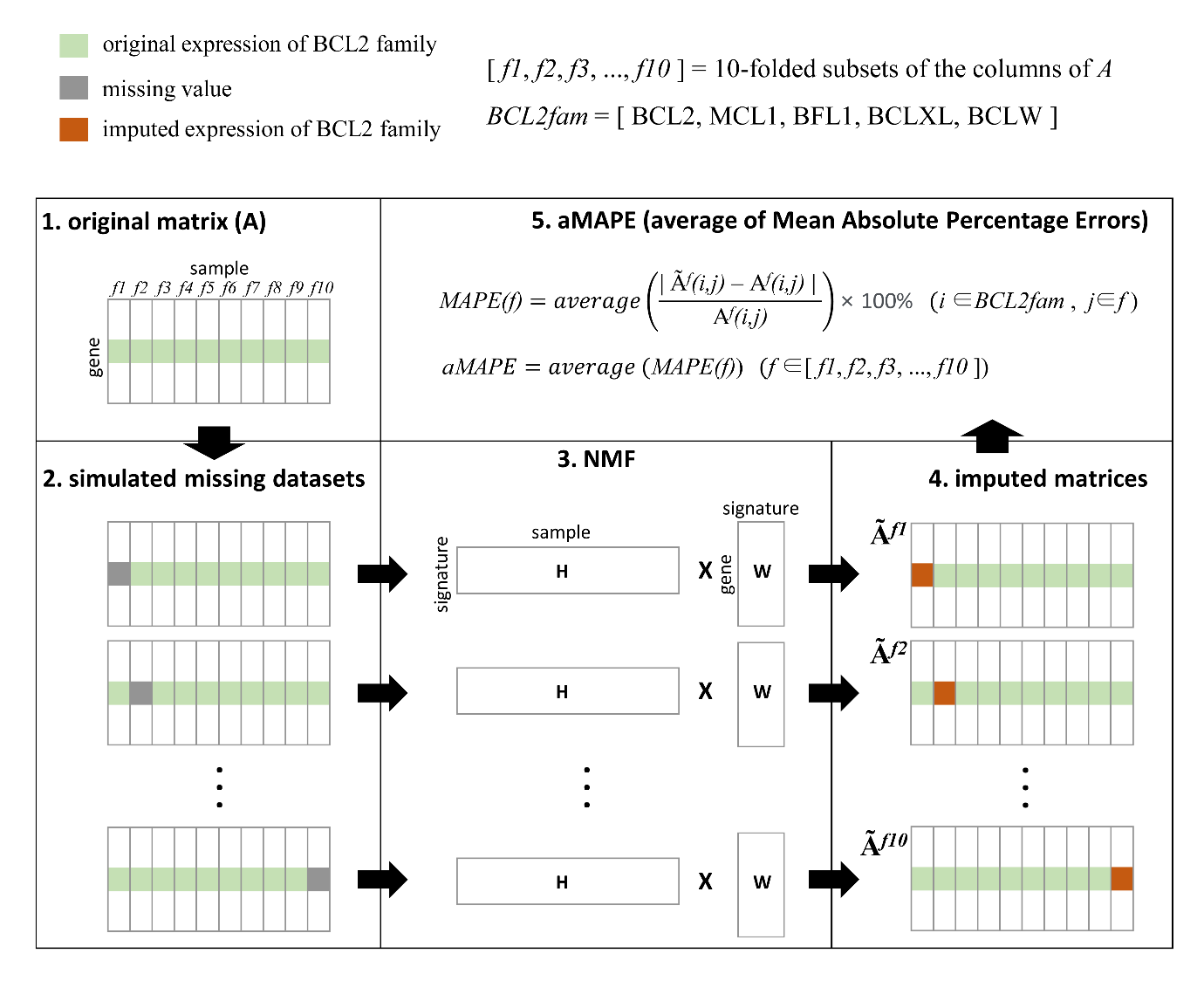


**Fig S20. Performance Measurements in Imputation of BCL2 family Profiles**

The average of mean absolute percentage errors (aMAPE) is calculated using intermediate gene sets during the gene optimization process. (1) In the expression matrix of the given gene set, the samples (columns) are split into 10-folds. (2) For each fold, the expression of BCL2 family genes is set to missing values (*NA* value in R), resulting in 10 simulated missing datasets. (3-4) Non-negative matrix factorization (NMF) is applied to the simulated datasets and their result matrices are multiplied to produce imputed matrices. (5) Finally, aMAPE is calculated by comparing the imputed and original BCL2 family expression.
